# The Biological Roles and Clinical Applications of the PI3K/AKT Pathway in Targeted Therapy Resistance in HER2-Positive Breast Cancer: A Comprehensive Review

**DOI:** 10.3390/ijms252413376

**Published:** 2024-12-13

**Authors:** Hanyi Zhong, Ziling Zhou, Han Wang, Ruo Wang, Kunwei Shen, Renhong Huang, Zheng Wang

**Affiliations:** Department of General Surgery, Comprehensive Breast Health Center, Ruijin Hospital, Shanghai Jiao Tong University School of Medicine, Shanghai 200025, China; zhonghanyi2010@163.com (H.Z.); yugizzl@hotmail.com (Z.Z.); wanghan8844@163.com (H.W.); wangruo@sjtu.edu.cn (R.W.); kwshen@medmail.com.cn (K.S.)

**Keywords:** HER2-positive breast cancer, targeted therapy, PI3K/AKT pathway, drug resistance

## Abstract

Epidermal growth factor receptor 2-positive breast cancer (HER2+ BC) is a highly invasive and malignant type of tumor. Due to its resistance to HER2-targeted therapy, HER2+ BC has a poor prognosis and a tendency for metastasis. Understanding the mechanisms underlying this resistance and developing effective treatments for HER2+ BC are major research challenges. The phosphatidylinositol-3-kinase/protein kinase B (PI3K/AKT) pathway, which is frequently altered in cancers, plays a critical role in cellular proliferation and drug resistance. This signaling pathway activates various downstream pathways and exhibits complex interactions with other signaling networks. Given the significance of the PI3K/AKT pathway in HER2+ BC, several targeted drugs are currently in development. Multiple drugs have entered clinical trials or gained market approval, bringing new hope for HER2+ BC therapy. However, new drugs and therapies raise concerns related to safety, regulation, and ethics. Populations of different races and disease statuses exhibit varying responses to treatments. Therefore, in this review, we summarize current knowledge on the alteration and biological roles of the PI3K/AKT pathway, as well as its clinical applications and perspectives, providing new insights for advancing targeted therapies in HER2+ BC.

## 1. Introduction

Breast cancer (BC) is one of the most prevalent cancers worldwide, representing the second leading cause of cancer-related mortality among women globally [1]. Clinically, breast cancer is categorized into the three following major subtypes based on the expression of estrogen receptor (ER), progesterone receptor (PR), and human epidermal growth factor receptor 2 (HER2, also known as ERBB2): hormone receptor-positive breast cancer (HR+ BC), HER2-positive breast cancer (HER2+ BC), and triple-negative breast cancer (TNBC) [2]. Approximately 20% to 25% of breast cancer cases exhibit HER2 overexpression, which is characterized by a higher propensity to display metastasis, resistance to chemotherapy and hormonal therapy, shortened survival, and poor prognosis.

**Figure 1:**
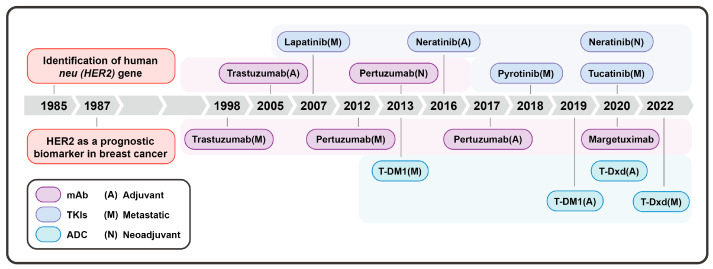
The pathway of drugs targeting HER2 for the treatment of HER2+ breast cancer.

HER2-targeted therapy focuses on HER2, disrupting its signaling pathways through two primary mechanisms: utilizing extracellular domain (ECD) antibodies to prevent receptor dimerization and employing tyrosine kinase inhibitors (TKIs) to inactivate the catalytic activity and abolish phosphorylation. These measures inhibit HER2 signaling and exert antitumor effects (Figure 1). This approach has substantially improved the pathologic complete response (pCR) rates in early-stage breast cancer (eBC), and has also improved the progression-free survival (PFS) and overall survival (OS) in metastatic cases, making it an important strategy for improving outcomes in patients with HER2-positive breast cancer [3].

Based on the breast cancer treatment guidelines, the first-line targeted therapy for HER2+ BC consists of monoclonal antibodies, with trastuzumab and pertuzumab acting as key representatives. These agents, respectively, bind to the extracellular domains IV and II of the HER2, interfering with the binding of the epidermal growth factor receptor (EGFR) to HER2 and preventing the formation of heterodimers. Additionally, they engage with the Fc receptors of immune effector cells, inducing antibody-dependent cellular cytotoxicity (ADCC). They have become the standard treatment for HER2+ BC [4].

Second-line therapies include antibody–drug conjugates (ADCs) such as trastuzumab emtansine (T-DM1) and trastuzumab deruxtecan (T-Dxd). ADCs are formed by conjugating cytotoxic drugs to monoclonal antibodies via linkers; they can achieve more precise drug delivery and enhanced therapeutic effects through the bystander effect [5].

Third-line interventions and later treatments involve the use of margetuximab and TKIs. TKIs are small molecules capable of entering the cell and competitively inhibiting the ATP-binding domain (tyrosine kinase) of HER2, thus preventing autophosphorylation and inhibiting HER2-mediated downstream signaling, ultimately exerting antitumor effects. TKIs are particularly beneficial in overcoming the issue of it being difficult for monoclonal antibodies to cross the blood–brain barrier. The TKIs currently used in breast cancer treatment include lapatinib, neratinib, tucatinib, and pyrotinib.

Despite the significant improvements in patient outcomes achieved with anti-HER2 therapies, both as monotherapies and in combination, treatment resistance remains a major challenge. While many patients initially demonstrate favorable responses, the majority eventually experience reduced therapeutic efficacy, disease progression, and metastasis [6]. This not only negatively affects patient survival, but also presents significant challenges in clinical management. In advanced stages of the disease, 50% of patients experience central nervous system (CNS) recurrence [7]. Furthermore, among metastatic breast cancer (MBC) patients treated with TKIs, approximately 20% develop resistance within one year. These challenges are compounded by molecular biological alterations that can impact treatment efficacy across different stages of therapy. This complicates disease management and significantly elevates mortality risk, underscoring the need for a deeper understanding of the resistance mechanisms.

The mechanisms of resistance to anti-HER2 targeted therapies are multifaceted, involving the regulation of signaling pathways and genetic mutations. Because all three drugs target HER2, there are shared mechanisms of resistance that have been identified. First, some tumor cells may develop resistance by increasing HER2’s expression or altering its structure, such as through deletions of ECD. Mutations and aberrant expression of downstream HER2 signaling pathways can also induce resistance. Other cells bypass the inhibitory effects of therapy by activating alternative signaling pathways. Moreover, intracellular changes, such as the regulation of miRNAs and mutations in other oncogenes, may promote resistance. These changes can inhibit drug internalization and lysosomal processing, and the presence of drug efflux pumps has been confirmed [8]. Also, changes in the tumor microenvironment may limit the effectiveness of the drugs.

Therefore, understanding these resistance mechanisms is crucial for developing more effective treatment strategies. The main challenges in HER2-targeted therapy lie not only in prolonging the efficacy of the drugs but also in overcoming resistance through the use of innovative approaches. Several new therapies have already found use in preclinical and clinical applications, including combinations of various targeted therapies, the use of immunotherapy, and the application of precision medicine. One of the key factors contributing to resistance is the dysregulation of intracellular signaling pathways, particularly the phosphoinositide 3-kinase (PI3K)/AKT pathway, which is often activated in HER2+ BC. PI3K/AKT signaling plays a central role in mediating cellular survival, proliferation, and metabolism, and its activation can bypass the HER2 signaling blockade, rendering therapies less effective. This review will focus on the mechanisms by which the PI3K/AKT pathway contributes to resistance to HER2-targeted therapies, and will also focus on the PI3K/AKT inhibitors currently under investigation. The aim is to address the challenges faced by HER2-positive breast cancer patients and provide more durable and personalized treatment options.

## 2. PI3K/AKT Pathway and HER2+ Breast Cancer

### 2.1. PI3K/AKT Pathway in HER2-Driven Breast Cancer

HER2 belongs to the EGFR family and is a type of receptor tyrosine kinase (RTK) [9]. When a ligand binds to the receptor, HER2 forms heterodimers with other members of the EGFR family, leading to phosphorylation. Phosphorylated receptors can activate downstream signaling pathways, including the PI3K/AKT/mammalian target of rapamycin (mTOR) (PAM), Ras/Raf/MEK/signal-regulated kinase (ERK) 1/2, and phospholipase C (PLCγ) pathways [10]. These regulate cell growth, tumor proliferation, and apoptosis. HER2+ BC can be classified into three subtypes based on HER2 status: HER2-amplified breast cancer, HER2-mutant breast cancer, and ligand-activated HER2 breast cancer. The majority of cases involve the amplified type [11]. In HER2+ BC cells, HER2 amplification disrupts the balance between cell proliferation and apoptosis, significantly enhancing tumor cell survival and promoting the initiation and progression of cancer.

The PI3K/AKT pathway is one of the two critical downstream signaling pathways of HER2, and HER2 signaling is primarily transmitted through the PI3K/AKT/mTOR pathway, regulating cell proliferation, growth, metabolism, and cell death. Previous studies have confirmed that this pathway is one of the most dysregulated pathways in cancer, playing a role in the development of various cancers, and it has been particularly studied in breast cancer, especially in HR+ BC. In recent years, increasing attention has been paid to its function in HER2+ BC [12]. A study has shown that PI3K is involved in the tumorigenesis driven by HER2, with experimental evidence indicating that HER2 can activate the PI3K pathway directly or indirectly, and that mutations in the sites mediating the interaction between PI3K and HER2 can reduce tumorigenicity [13].

Additionally, the PI3K/AKT pathway can upregulate immunosuppressive genes or promote the expression of genes associated with metastasis, such as PD-L1, cytotoxic T-lymphocyte-associated antigen-4, colony stimulating factor 1 (CSF1), and CSF1 receptor, in cancer cells or microglia within the brain metastasis microenvironment. PI3Kγ signaling in tumor-associated macrophages (TAMs) has been shown to support tumor growth by promoting immunosuppression in head and neck squamous cell carcinoma. In mouse models of breast cancer, CSF1 signaling in TAMs has been demonstrated to enhance the invasiveness and infiltration of breast cancer cells in vivo, and inhibiting this signaling can suppress tumor growth by reducing TAM numbers and increasing CD8+ T-cell infiltration [14].

The two most important components of the PI3K/AKT pathway are PI3K and AKT. PI3K possesses both serine/threonine kinase and phosphatidylinositol kinase activities and is composed of a catalytic subunit (p110) and a regulatory subunit (p85) [15]. PI3K is activated by the binding of ligands (such as insulin, growth factors, and hormones) to RTKs or G protein-coupled receptors, converting phosphatidylinositol-4,5-bisphosphate (PIP2) into phosphatidylinositol-3,4,5-trisphosphate (PIP3). AKT is then recruited to the plasma membrane, where it undergoes two phosphorylation events: the first is catalyzed by phosphoinositide-dependent protein kinase 1 (PDK1) as part of a threonine residue, and the second is stimulated by mTORC2 [16].

The PI3K/AKT pathway is highly regulated by external factors and exhibits extensive crosstalk with other pathways. In HER2+ BC cells, once phosphorylated and activated, AKT is distributed to the cytoplasm and nucleus, where it phosphorylates other substrates and activates downstream pathways, building networks related to cell proliferation and survival. Studies have shown that the activation of the PI3K/AKT pathway increases the phosphorylation of IKKα, Chk1, p21, p27, glycogen synthase kinase 3 (GSK3), and tuberous sclerosis 2 (TSC2), promoting cell survival and proliferation and enhancing cellular metabolism. It also inhibits apoptosis by phosphorylating forkhead box O (FOXO) to suppress its transcriptional activity, increasing the phosphorylation of MDM2 to inhibit p53, increasing BAD phosphorylation, and upregulating the expression of the anti-apoptotic proteins Bcl-2 and Bcl-xL.

The phosphatase and tension homolog (PTEN) is a major negative regulator of the PI3K pathway. The C2 domain of PTEN facilitates its localization to the cell membrane, and PTEN has an affinity for phospholipid membranes in vitro. Nuclear PTEN is critical for maintaining chromosomal integrity and centromere stability. As a lipid phosphatase, PTEN negatively regulates the PI3K pathway by converting PIP3 back to PIP2. When PTEN is mutated or inactivated, PI3K/AKT signaling is activated, even in the absence of exogenous oncogenic stimuli, and this leads to cellular transformation and tumorigenesis. One study shows that even slight reductions in PTEN expression significantly increase cancer susceptibility [17]. On the other hand, PTEN also participates in tumor signaling by dephosphorylating protein targets such as focal adhesion kinase (FAK), insulin receptor substrate 1, c-SRC, and PTEN itself. PTEN plays a critical role in controlling tumor cell migration and angiogenesis.

Beyond PTEN, SH2-containing 5′ inositol phosphatases (SHIP1/2) and inositol polyphosphate-4-phosphate type II B (INPP4B) also negatively regulate the PI3K/AKT pathway [18]. SHIP1/2 dephosphorylates PIP3 to phosphatidylinositol-3,4-bisphosphate (PI(3,4)P2), and PI(3,4)P2 is dephosphorylated by INPP4B to become phophatidylinositol-3-phosphate [19]. The overexpression of INPP4B inhibited cell proliferation, migration, and EGF-induced epithelial–mesenchymal transition (EMT) progression [20]. INPP4B depletion antagonized the inhibitory effects of HER2 depletion on the proliferation and migration of HER2+ BC cells. INPP4B was thus proposed as a tumor suppressor protein, and it was proved to enhance the antitumor effects of lapatinib [21].

### 2.2. Alterations of PI3K/AKT Pathway in HER2+ Breast Cancer

Cells and tumors depend on the PI3K pathway in multiple ways, with alterations in most of the components of this network [22]. This includes PIK3CA and AKT gene mutations, as well as the loss of the tumor suppressor PTEN [23]. In HER2+ BC, alterations in the PI3K/AKT pathway play a significant role in tumor progression. Among patients receiving anti-HER2 therapy, 44% exhibit mutations in genes involved in the PI3K pathway [24] (Table 1).

The PIK3CA mutation is the most common alteration in the PI3K pathway in HER2+ BC. The PIK3CA mutation rate in HER2+ BC ranges from 12% to 39%, and the frequency of PIK3CA mutations varies significantly depending on HR status and HER2 expression [31]. A retrospective study that pooled five cohorts of HER2+ BC patients found a PIK3CA mutation rate of 21.7% (21.4% in the GeparStudies, 22.5% in NeoALTTO, and 20.4% in the CHERLOB study). It identified hotspot mutations in the PIK3CA gene (H1047R, E545K and E542K), with a mutation rate of 15.2% in HER2+ BC [32]. Another study analyzed data from 6338 patients with BC across 10 publicly available studies. Among these patients, 31% of the HER2+ BC cases had PIK3CA mutations. It was found that the PIK3CA mutations were highly heterogeneous, with five mutations representing ~ 70% of all the known types of PIK3CA mutations in the dataset, including H1047R (35%), E545K (17%), E542K (10%), N345K (2%), and H1047L (5%) [33]. PIK3CA mutations, HR status, and treatment interact, affecting outcomes such as the pCR and PFS [34]. In studies on HER2+ MBC, the inhibition of the PI3K pathway and its downstream pathways have been shown to improve PFS in tumors with PIK3CA mutations [35]. Also, whole-genome expression analysis found that the downregulation of the PI3K pathway may be a positive predictive factor for the disease [36].

However, alterations in the PI3K pathway alone cannot predict survival in early-stage or metastatic HER2+ BC [37]. In patients treated with the pan-TKI pyrotinib, single-gene mutations, including PIK3CA mutations, did not show significant predictive or prognostic value. However, patients with multiple gene alterations had significantly shorter median PFSs compared to those with no or only one gene mutation, suggesting crosstalk between the PI3K pathway and various other pathways. The changes could involve HER2 bypass signaling pathways, the PAM pathway, and genetic variations in TP53, including single-nucleotide variants and copy number variants [38].

AKT is another critical component of the PI3K/AKT pathway. AKT alterations manifest as AKT gene mutations and, more frequently, AKT amplifications. In the TCGA breast cancer cohort, 17.76% of the cases (195/1098) involved 207 AKT alterations (*p* = 0.0002). Among these, AKT3 mutations were the most common, with a mutation rate of 14.2%, followed by changes in AKT2 and AKT1. The alterations in the TCGA cohort included 189 cases of amplification, primarily found in relation to AKT3 (91.3%); 8 cases of missense mutations (3.9%); 7 cases of homozygous deletions (HOMDEL) (3.4%); 1 case of synonymous mutation (0.5%); and 2 cases of nonsense mutations (1.0%) [25]. In a Chinese breast cancer cohort, researchers found that most of the missense mutations were AKT1 E17K mutations, a finding not observed in the TCGA cohort [39]. Current research suggests that the AKT1 E17K mutation may play both antitumor and oncogenic roles in tumor cells [40]. Although AKT mutations play a significant role in ER+ and PR+ tumors, little is known about their role in HER2+ BC, particularly given that AKT1 mutations are only observed in tumors co-expressing ER and PR [41]. Therefore, further studies are required to understand the relationship between HER2 expression and AKT activity in different populations.

In normal cells, negative regulators such as PTEN keep PI3K/AKT signaling inactive, maintaining the balance between cell survival and apoptosis. However, in tumor cells, these negative regulators are dysfunctional, and the PI3K/AKT pathway remains abnormally activated, continuously promoting cell motility, survival, and proliferation while inhibiting apoptosis.

PTEN mutations occur in <2% of breast cancers [42]; however, the loss of the PTEN protein occurs in approximately 48% of cases [29]. A study of a continuous cohort of Chinese patients with primary breast cancer showed that the mutation rate of the PTEN gene was 0.23%, suggesting that germline mutations of the PTEN gene may not be closely related to the incidence of breast cancer in the Chinese population [43]. However, growing evidence indicates that alterations in PTEN are associated with tumor initiation, metastasis, and other aspects of disease progression.

PTEN mutations include the deletion of a single-gene copy of PTEN and gene silencing, as well as mutations of the PTEN coding sequence [44]. PTEN deletion activates the NF-κB pathway, increasing the expression of the chemokine CCL2 in breast cancer cells. CCL2-positive cancer cells recruit macrophages that express the CCL2 receptor and, following cancer cell extravasation, promote the growth of brain metastases [14]. There is also evidence that the methylation of the PTEN promoter and miRNAs play roles in suppressing PTEN expression in certain types of tumors. PTEN deletion has been detected in 25% to 71% of brain metastases in breast cancer patients. Due to its epigenetic regulation by miRNAs in astrocytes, PTEN’s expression is often not easily detected in MBC cells in other organs [45]. Reduced PTEN expression is significantly associated with poorer OS in breast cancer patients lacking HR, with or without HER2 overexpression/amplification. It is PTEN protein expression, rather than genetic alterations, that is associated with OS and PFS [46].

Compared to PIK3CA mutations, the loss of PTEN is more strongly correlated with poor prognosis in ER-negative invasive breast cancer, and patients with both PTEN deletion and PIK3CA mutations have a higher risk of death [47]. Additionally, the transcriptional product of the PTEN pseudogene (PTENP1) also regulates PTEN. PTENP1 contains miRNA binding sites on its 3′ UTR, similar to those of PTEN, and is thus considered a miRNA sponge, playing a critical role in various cancers, including breast cancer.

In addition to familial or somatic genetic mutations that lead to relatively stable oncogenes, tumors also evolve mechanisms at the mRNA and protein levels, regulating the expression of PI3K/AKT, promoting tumor growth, and inducing resistance. Several factors contribute to the regulation of the expression of PI3K/Akt/mTOR signaling components, including DNA methylation, histone modifications, chromatin remodeling, and regulation by non-coding RNAs.

DNA methylation can lead to the decreased transcription of target genes, making DNA or promoter methylation more commonly observed in genes that suppress tumor functions [48]. A study of Chinese breast cancer patients showed a significant correlation between AKT1 methylation levels and HER2 status (*p* = 0.0249), with low methylation and suppressed expression of AKT1 being associated with HER2-negative tumors [49].

Cowden syndrome is closely associated with mutations in the PTEN gene, with 25–50% of patients developing breast cancer. Among these patients, only 5% have PTEN mutations, but approximately 40% show reduced levels or the absence of the PTEN protein. Studies suggest that this phenomenon is linked to hypermethylation of the PTEN promoter, leading to decreased PTEN mRNA expression. This is associated with poor prognostic factors, including ERBB2 overexpression, larger tumor size, and a higher histological grade [50].

At the transcriptional level, non-coding RNAs also regulate the PI3K/AKT pathway. In HER2+ BC, miR-18a-5p suppresses the activation of the PI3K/AKT pathway by targeting HER2, thereby inhibiting tumor progression [51]. MiR-221 has been shown to be upregulated in various cancers and promotes cell growth [52]. In breast cancer, this is associated with EMT [53]. Studies have found that miR-221 targets PTEN. This reduces its expression, thereby inhibiting apoptosis in HER2+ BC, promoting metastasis, and inducing trastuzumab resistance [54]. Additionally, the long non-coding RNA (LncRNA) ORLNC1 can upregulate PTEN in HER2+ BC by competitively binding with miR-296, inhibiting the proliferation of these tumor cells both in vitro and in vivo [55].

Although research on the role of non-coding RNAs in regulating the PI3K/AKT signaling pathway in HER2+ BC tumor cells is limited and lacks specificity, numerous studies have demonstrated their important regulatory roles, particularly in breast cancer and TNBC. Therefore, the functions of non-coding RNAs in this context need to be further explored; they hold significant potential as prognostic markers and therapeutic targets.

Therefore, oncogenic alterations in the PI3K pathway are associated with poor prognosis in HER2+ BC patients receiving HER2-targeted therapy. Further research is needed to investigate and elucidate the impact of the PI3K pathway on treatment efficacy and resistance in HER2+ BC.

### 2.3. Evidence of PI3K/AKT Alterations in Inducing Resistance to Anti-HER2 Therapy

PIK3CA mutations, AKT alterations, and the loss of the PTEN tumor suppressor can lead to the constitutive activation of the PI3K/AKT signaling pathway [56].

Studies have found that mutations in PIK3CA can induce resistance to trastuzumab [57] or combination anti-HER2 therapies [58] in breast cancer cells in vitro. In the context of neoadjuvant therapy for eBC, PIK3CA mutations are often associated with poorer prognosis in patients, including lower rates of pCR [28,59]. Due to the hyperactivation of the PI3K pathway, subclones with PIK3CA or ERBB2 hotspot mutations are preferentially selected in resistant HER2+ BC [60]. Patients who are sensitive to trastuzumab are less likely to harbor TP53 and PIK3CA mutations than those who are resistant. Moreover, in breast cancer patients, the presence of driver mutations, a higher tumor mutational burden, a greater copy number load, and an APOBEC signature significantly reduce the efficacy of targeted therapies [61]. Additionally, research has shown that T-DM1 is active in PIK3CA-mutant breast cancer cell lines and xenograft models, likely due to its unique mechanism of action, which involves the HER2-mediated delivery of a chemotherapeutic payload, independent of downstream HER2 signaling [62].

PTEN deletion is also associated with poor outcomes in anti-HER2 therapy. Approximately 20% to 25% of HER2+ BC cases exhibit a significant loss of PTEN expression, and most activating PIK3CA mutations occur in PTEN-deficient cases. Assessing the expression levels of both genes simultaneously can help to predict responses to trastuzumab [57]. In the absence of PTEN, PI3Kβ inactivation reduces signal transducer and activator of transcription 3 (STAT3) signaling and increases the expression of immunostimulatory molecules, thereby promoting antitumor immune responses. This suggests that PI3Kβ regulates immune evasion in PTEN-deficient tumors through STAT3 signaling [63].

A study found that, in trastuzumab-resistant HER2+ BC patients, PTEN loss and PIK3CA mutations commonly result in the activation of the PI3K pathway [64]. When PIK3CA mutations co-occur with PTEN mutations, the pCR rate in PTEN-low/PIK3CA-mutant tumors is further reduced compared to that in PTEN-high/PIK3CA-mutant tumors [65].

## 3. The Role of the PI3K/AKT Pathway in Resistance to Anti-HER2 Therapy in HER2+ Breast Cancer

### 3.1. PI3K/AKT and Its Downstream Signaling Pathways

**Figure 2:**
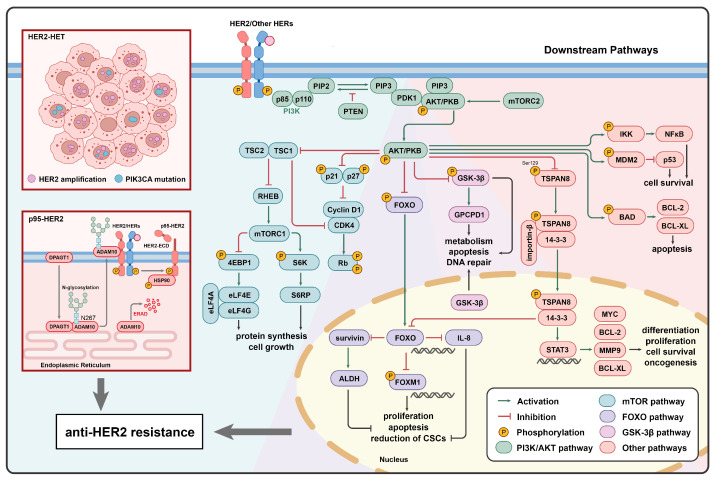
Function of the PI3K/AKT pathway and its involvement in anti-HER2 treatment resistance. HER2, human epidermal growth factor receptor 2; PI3K, phosphoinositide 3-kinase; HER2-HET, HER2-heterogeneous; DPAGT1, dolichyl-phosphate N-acetylglucosaminephosphotransferase 1; ECD, extracellular domain; PIP2, phosphatidylinositol-4,5-bisphosphate; PIP3, phosphatidylinositol-3,4,5-trisphosphate; PTEN, phosphatase and tension homolog; mTORC, mammalian target of rapamycin complex; TSC, tuberous sclerosis complex; 4EBP1, eukaryotic translation initiation factor 4E-binding protein 1; S6K, ribosomal protein S6 kinase; FOXO, forkhead box O; FOXM1, forkhead box protein M1; ALDH, aldehyde dehydrogenase; IL-8, interleukin-8; CSC, cancer stem cell; GSK-3β, glycogen synthase kinase 3 beta; GPCPD1, glycerophosphodiesterase 1; TSPAN8, tetraspanin-8; STAT3, signal transducer and activator of transcription 3; IKK, inhibitor of kappa B kinase; MDM2, murine double minute 2; BAD, BCL2-associated agonist of cell death; Bcl-2, B-cell lymphoma 2; BCL-XL, B-cell lymphoma-extra-large; MYC, myelocytomatosis oncogene; MMP9, matrix metalloproteinase-9.

Based on the transmission of HER2 signaling, the PI3K/AKT pathway plays a crucial role in influencing the efficacy of anti-HER2 therapies. PI3K/AKT signaling, activated by HER2/HER3 dimerization, is one of the most critical survival pathways downstream of HER2. The aberrant activation of the PI3K pathway significantly contributes to the development of resistance to anti-HER2 therapies [66]. The PI3K/AKT pathway is regulated by upstream HER2, while simultaneously activating several downstream signaling pathways. In HER2+ BC cells, the activation or alteration of these pathways plays a vital role in various cellular processes, including proliferation, apoptosis, and glucose metabolism (Figure 2).

#### 3.1.1. Regulation of PI3K/AKT by HER2 Alterations

The proteolytic cleavage or shedding of HER2 leads to the release of ECD epitopes and generates constitutively active intracellular fragments (p95-HER2), a structural alteration observed in approximately 30% of HER2+ BC cases. High levels of expression of p95-HER2 can induce resistance to anti-HER2 therapies by activating the PI3K/AKT signaling pathway [67]. p95-HER2 is characterized by a truncated ECD, and so it lacks the epitope required for trastuzumab binding. This impairs the effectiveness of anti-HER2 therapies, particularly those involving monoclonal antibodies. However, studies have shown that tumors expressing p95-HER2 can respond to TKIs. Both full-length versions and p95-HER2 interact with the HSP90 chaperone protein. The loss of p95-HER2 expression has been associated with the downregulation of the PI3K/AKT and extracellular ERK pathways, as well as the inhibition of cell proliferation. In trastuzumab-resistant models lacking p95-HER2 expression, prolonged treatment with HSP90 inhibitors in vivo results in the sustained loss of both HER2 and p95-HER2, the inhibition of AKT activation, the induction of apoptosis, and the complete suppression of tumor growth [68]. These findings highlight the role of HSP90 as a crucial regulator of p95-HER2 expression and function, while the PI3K/AKT pathway plays a pivotal role in mediating the trastuzumab resistance induced by p95-HER2.

The mechanism of HER2 ligand shedding has also been investigated. Following trastuzumab treatment, the upregulation of the protease ADAM10 leads to HER2 ligand shedding [69], and this process is associated with reduced phosphorylation of AKT/PKB [70]. Dolichyl-phosphate N-acetylglucosaminephosphotransferase 1 (DPAGT1) is a key component of the N-glycosylation process [71], and the inhibition of DPAGT1 by the glycosylation inhibitor tunicamycin has been shown to resensitize trastuzumab-resistant HER2+ breast tumors. Membrane-bound DPAGT1 is internalized via the caveolar pathway and retrogradely transported to the endoplasmic reticulum. DPAGT1 induces the N-glycosylation of ADAM10 at N267, protecting it from endoplasmic reticulum-associated degradation (ERAD) and mediating HER2 shedding. This results in the upregulation of the downstream PI3K/AKT pathway and contributes to the development of trastuzumab resistance [72]. In liver cancer, tunicamycin was found to reverse cisplatin resistance by inhibiting the DPAGT1/AKT/ABCG2 pathway [73], and the co-administration of tunicamycin with trastuzumab has been shown to enhance antitumor efficacy [74]. These findings further elucidate the relationship between DPAGT1, ADAM10, and AKT in the development of cancer drug resistance.

Additionally, as HER2+ BC is a heterogeneous disease, there are biological differences between HER2-heterogeneous (HET) tumors and non-HET tumors [75]. Clinical trials have shown that HER2-HET tumors exhibit reduced responses to ADC-targeted therapies, potentially due to decreased HER2 protein levels. This reduces the T-DM1 binding affinity, thereby diminishing treatment efficacy. Studies of T-DM1+pertuzumab therapy in HER2+ BC have shown that HET/non-pCR patients express higher levels of PI3K pathway components. HER2-HET tumors maintain elevated PI3K/ERBB signaling through the upregulation of downstream pathway components [76]. Therefore, in patients with HET tumors, targeted therapies using small-molecule inhibitors of EGFR family members and downstream components of the PI3K signaling pathway may be more effective.

#### 3.1.2. The PI3K/AKT/mTOR Pathway

The hyperactivation of the PAM pathway, one of the most important signaling pathways downstream of PI3K/AKT, leads to HER2-dependent mTOR pathway activation, resulting in HER2 resistance.

The phosphorylation of the effector protein eukaryotic translation initiation factor 4E-binding protein 1 (4EBP1) within the PAM pathway is associated with poor prognosis in breast cancer and other malignancies due to the presence of elevated p4EBP1 levels in tumors [77]. p4EBP1 activation regulates protein synthesis via eIF4E, forming the eIF4E–eIF4G–eIF4A complex, which produces new proteins following lapatinib treatment and diminishes its anti-proliferative activity. PIK3CA mutations, PTEN, and 4EBP1 serve as synergistic factors in different stages of this pathway [65]. In HER2+ BC cells with PIK3CA mutations, the attenuation of lapatinib’s effects by p4EBP1 is even more pronounced [24], suggesting that p4EBP1 has potential as a biomarker in terms of the efficacy of anti-HER2 therapies.

Cyclin D1/CDK4 acts as a key effector of AKT, mediating resistance to HER2-directed therapies, and CDK4/6 inhibitors can resensitize resistant tumor cells [78]. The inhibition of CDK4/6 not only prevents Rb phosphorylation but also reduces TSC2 phosphorylation, which suppresses the phosphorylation of mTORC1 substrates such as 4EBP1 and the activity of S6K/S6RP. This alleviates the feedback inhibition of upstream epidermal growth factor receptor family kinases, thereby resensitizing tumors to HER2 blockade [79].

#### 3.1.3. The PI3K/AKT/FOXO Pathway

FOXO is a significant effector of the PI3K/AKT pathway and is negatively regulated by it [80]. The dysregulation of FOXO can lead to uncontrolled cell proliferation and DNA damage, contributing to tumorigenesis [81]. Trastuzumab can interfere with the enhanced dimerization of HER2/HER3, and when PI3K/AKT is inhibited, HER3 mRNA and protein can be upregulated through FOXO-dependent mechanisms, facilitating HER2/HER3 dimerization [82]. Consequently, clinical studies indicate that “dual HER2 blockade”, performed using trastuzumab in combination with lapatinib or pertuzumab, is more effective than monotherapy with lapatinib or trastuzumab [83].

AKT phosphorylates the FOXO transcription factor, inhibiting its function and preventing FOXO from entering the nucleus, ultimately inhibiting apoptosis [84]. Studies have shown that PI3K inhibition can activate specific FOXO-dependent genes, mediating cell cycle arrest in breast cancer cells [85]. Treatment with the pan-PI3K inhibitor XL147 in combination with trastuzumab reduces tumor cell proliferation and pAKT levels. XL147 can induce apoptosis in trastuzumab-resistant cells while decreasing the proportion of cancer stem cells (CSCs) in both in vitro and in vivo settings. The analysis of biomarkers, like mammosphere formation, ALDH activity, and interleukin-8 (IL-8) expression, indicates that XL147, rather than trastuzumab, reduces CSC markers. This may be related to the FOXO-mediated transcriptional repression of anti-apoptotic genes such as survivin (BIRC5) and CSC-related cytokines like IL-8 [86]. FOXO3a is the most studied member of the FOXO subfamily. Knocking down FOXO3a with siRNA upregulates IL-8 mRNA levels and promotes mammosphere formation. Conversely, downregulating survivin with siRNA reduces mammosphere formation and ALDH activity, suggesting that survivin plays a role in maintaining the CSC population. This indicates that blocking the PI3K pathway, along with the further inhibition of survivin, may reduce the presence of tumor stem cells and potentially prevent the development of acquired resistance to anti-HER2 therapies.

Research has shown that the transcription factor FOXM1 is a direct target of FOXO protein suppression and serves as an upstream regulator of drug transporter proteins [87]. The inactivation of FOXO and the overexpression of FOXM1 are associated with tumorigenesis and cancer progression. The cytostatic and cytotoxic effects of various anticancer drugs, including lapatinib, gefitinib, imatinib, and cisplatin, are mediated through the activation of FOXO3a and/or the inhibition of its target FOXM1. Additionally, FOXO proteins promote the expression of genes that are critical for drug efflux, DNA repair, and cell survival pathways in resistant cancers, thereby contributing to drug resistance [88].

#### 3.1.4. The PI3K/AKT/GSK3 Pathway

GSK-3β promotes tumor cell growth and facilitates EMT by influencing DNA damage repair, making breast tumor cells resistant to chemotherapy and targeted therapies [89]. This repair function interacts with the PI3K/AKT pathway. In the cytoplasm, GSK-3β can phosphorylate target proteins, triggering proteasomal degradation and promoting NF-κB activity, thereby reducing apoptosis. In the nucleus, GSK-3β phosphorylates several DNA repair factors, such as UNG2 and BP53, facilitating DNA repair. Pharmacological inhibition and siRNA knockdown studies have demonstrated that, when AKT is inhibited, the translocation of phosphatidylserine and DNA breaks rapidly increase, indicating that GSK-3β signaling is partially responsible for inducing apoptosis [90].

Meanwhile, the phosphorylation of AKT, mediated by GSK-3β and PTEN, is associated with cell viability, migration, and apoptosis, which may promote chemotherapy resistance in breast cancer. Additionally, GSK-3β can regulate cell viability and induce chemotherapy resistance through the PTEN/PI3K/AKT signaling pathway [91]. Recent studies have found that the PI3K/AKT/GSK-3β pathway can modulate choline metabolism in breast cancer patients by regulating the expression of glycerophosphodiesterase EDI3 (GPCPD1). Given that the inhibition of EDI3 can reduce in vitro cell viability and in vivo tumor growth in ER-/HER2+ cells resistant to HER2-targeted therapies, EDI3 may represent a potential novel therapeutic target worthy of further exploration [92].

These findings indicate that mutations in the PI3K/AKT pathway can elicit novel responses to anti-HER2 therapies through complex mechanisms, leading to the abnormal expression of effector proteins and consequently mediating the development of resistance. In HER2+ BC, alterations in HER2 or PI3K result in the abnormal activation of PI3K/AKT signaling. Even when the HER2 is effectively inhibited by targeted therapies, tumor cells can still maintain proliferation and survival through this pathway. AKT activation further suppresses tumor suppressors such as FOXO and GSK-3β, leading to the inhibition of apoptosis and enhanced cell survival. Additionally, AKT promotes the expression of anti-apoptotic genes like BCL-2 and BCL-XL via the activation of the NF-κB pathway, further increasing cell survival and drug resistance. Moreover, AKT enhances protein synthesis and cell growth by inhibiting the TSC1/TSC2 complex and activating mTORC1. This boosts the proliferative capacity of the tumor. AKT also accelerates cell cycle progression by regulating cell cycle control factors such as p21 and Cyclin D1, further driving tumor cell proliferation. As a result, while current anti-HER2 therapies effectively inhibit HER2 signaling, the PI3K/AKT pathway and its downstream effects, including cell cycle regulation, apoptosis inhibition, and protein synthesis, remain active, contributing to the development of resistance.

### 3.2. Interaction of the PI3K/AKT Pathway with Other Signaling Pathways

**Figure 3:**
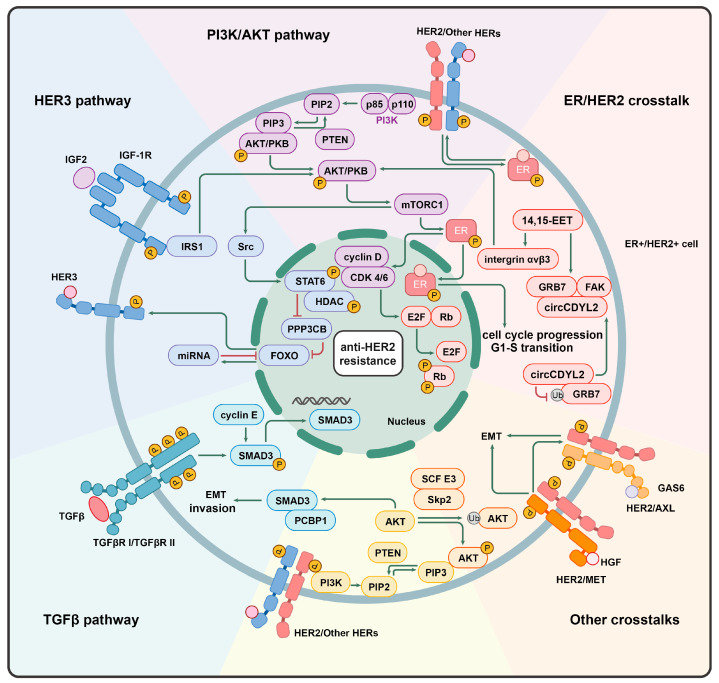
The role of the PI3K/AKT pathway in the resistance to anti-HER2 therapy with crosstalk of other signaling pathways. HER2, human epidermal growth factor receptor 2; PI3K, phosphoinositide 3-kinase; PIP2, phosphatidylinositol-4,5-bisphosphate; PIP3, phosphatidylinositol-3,4,5-trisphosphate; PTEN, phosphatase and tension homolog; mTORC, mammalian target of rapamycin complex; CDK4/6, Cyclin-dependent kinases 4 and 6; ER, estrogen receptor; 14,15-EET, 14,15-Epoxyeicosatrienoic acid; GRB7, growth factor receptor-bound protein 7; FAK, focal adhesion kinase; Rb, retinoblastoma protein; AXL, AXL receptor tyrosine kinase; EMT, epithelial–mesenchymal transition; GAS6, growth arrest-specific 6; IGF-1R, insulin-like growth factor 1 receptor; IRS1, insulin receptor substrate; HER3, human epidermal growth factor receptor 3; STAT6, signal transducer and activator of transcription 6; HDAC, histone deacetylase; PPP3CB, protein phosphatase 3 catalytic subunit beta; FOXO, forkhead box O; TGFβ, transforming growth factor-beta; SMAD3, mothers against decapentaplegic homolog 3; PCBP1, Poly (C)-binding protein 1; SCF, Skp, Cullin, F-box-containing complex; Skp2, S-phase kinase-associated protein 2.

PI3K/AKT pathway interacts with numerous signaling pathways. Tumor cells that develop resistance to HER2-targeted therapies activate other compensatory pathways. Signals from other ERBB/HER can trans-activate HER2 and amplify downstream signaling, thereby circumventing the inhibitory effects of targeted therapies. The upregulation of RTKs such as HER3, HGF/c-MET, AXL, and FGFR enhances the PI3K/AKT/mTOR anx Src pathways, promoting tumor cell proliferation (Figure 3).

#### 3.2.1. HER3

The HER2/HER3 heterodimer is frequently implicated in tumor initiation and development, and the formation of this heterodimer is one of the characteristics of HER2’s role in tumor amplification [13]. In HER2+ BC with brain metastases, resistance to PI3K inhibitors caused by HER2 amplification and PIK3CA mutations can be alleviated by inhibiting HER3 activity both in vitro and in vivo, indicating that HER3 promotes PI3K-mediated brain metastasis [93].

Moreover, the HER3 E928G mutation in the kinase domain enhances the affinity of the HER2/HER3 dimer and reduces HER2’s binding to inhibitors such as neratinib. The co-expression of mutant HER2/HER3 intensifies the ligand-independent activation of HER2/HER3 and the PI3K/AKT pathways. This contributes to increased tumor growth, invasiveness, and resistance to HER2-targeted therapies. Combination treatment with PI3Kα inhibitors can reverse this resistance [94]. The pharmacological inhibition of HER3 can sensitize breast cancer cells to lapatinib, both in vitro and in vivo, by reducing the levels of phosphorylated HER3, HER3, and phosphorylated AKT [95].

Drug therapies targeting HER2 promote the expression of HER3 in breast cancer cells. The overexpression of HER3 can activate the PI3K/AKT pathway, and upregulation of activated HER3 limits the inhibitory effects of TKIs [96]. This upregulation is dependent on the activation of the PI3K/AKT pathway and its regulation of nuclear FOXO3a protein. The activation of IGF2/IGF-1R/IRS1 signaling is one of the key mechanisms underlying anti-HER2 resistance in breast cancer, with this activation occurring through the AKT/mTOR pathway, which in turn activates Src kinase. The Src kinase mediates the phosphorylation of STAT6 and HDAC1, collectively inhibiting the transcription of serine/threonine-protein phosphatase 2B catalytic subunit beta (PPP3CB). This results in elevated phosphorylation of the FOXO family member FOXO3a, disrupting the FOXO3a–miRNA negative feedback loop and causing the aberrant activation of the IGF2/IGF-1R/IRS1 pathway [97].

Meanwhile, when FOXO is activated, cell survival mechanisms are induced through feedback at various levels in the PI3K/AKT/FOXO pathway, including feedback from RTK and PI3K signaling, leading to enhanced FOXO transcription [98]. AKT-mediated negative feedback signaling increases the transcriptional activity of FOXO3a, driving the upregulation of HER3 expression. The increased HER3 is phosphorylated by HER2, while the reduction in the phosphatase activity of HER3 hinders dephosphorylation, thereby maintaining the PI3K/AKT pathway’s activity [96] and compensating for tumor growth signaling.

Recently, researchers found that, during continuous treatment with TKIs, dormant HER2+ BC cells gradually transitioned into proliferating drug-resistant BC cells [99]. Momeny Majid et al. conducted transcriptomic analysis of the cells during this process and discovered that the expression of the HER3 activator DUSP6 was suppressed in dormant cells but increased in proliferating cells. DUSP6 mediates resistance to HER2-targeted therapy through the neuroglial protein–HER3 axis. The inhibition of DUSP6 reduced HER3 expression, decreasing tumor lethality and tumor growth in brain metastasis models [100]. This indicates that the interaction between HER2 and HER3 plays a significant role in resistance to anti-HER2 therapy and is regulated by multiple factors [101].

#### 3.2.2. TGFβ

TGFβ is a multifunctional cytokine expressed in nearly all tissue and cell types, stimulating various cellular responses. The dysregulation of TGF-β can contribute to the progression of multiple cancers [102]. In the context of HER2 overexpression, ERK and p38 MAPK, mediate TGFβ-induced migration and invasion of breast epithelial cells via the PI3K/AKT pathway [103]. Previous studies have confirmed that HER2/EGFR activity is essential for the TGFβ-stimulated migration of breast cancer cells, while the inhibition of HER2/EGFR activity can enhance the inhibitory effects of TGFβ on cellular processes.

Trastuzumab stimulates ADCC, leading to the release of IFN and TGFβ. It also induces a high level of expression of PDL1 in tumor cells, which mediates resistance to trastuzumab [104]. SMAD3 is an effector protein in TGFβ signaling [105], and the persistent activation of the TGFβ-SMAD3 pathway induces resistance to anti-HER2 drugs and enhances the CSC characteristics of HER2+ BC cells. The nuclear expression of SMAD3 in tumor tissues correlates inversely with sensitivity to neoadjuvant trastuzumab treatment. Activated SMAD3 regulates multiple genes containing SMAD-binding elements that contribute to trastuzumab resistance [106]. The HER2/EGFR pathway stimulates the phosphorylation of Smad3 at the S208 site through PI3K/AKT, enhancing SMAD3 activity and increasing SMAD3’s nuclear accumulation [107]. The TGFβ and HER2/EGFR pathways collaboratively promote the EMT by regulating the interaction between SMAD3 and PCBP1, and CD44 splicing was also found to be involved in this process [108]. In HER2+ BC, the overexpression of cyclin E also induces trastuzumab resistance through the phosphorylation of SMAD3 [109].

Guoxu Zheng et al. found that the treatment of tumor cells and NK cells with trastuzumab leads to abnormal cytokine production, including TGFβ and IFN-γ. This subsequently enhances HLA-G/KIR2DL4 signaling. At the same time, TGFβ upregulates the PD-L1 on tumor cells and PD-1 on NK cells, impairing the cytotoxicity of NK cells [110]. This confirms the role of TGFβ in the mechanism of resistance to HER2-targeted therapy. This immune regulation mechanism provides new insights into the resistance mechanisms of trastuzumab and highlights the potential of combining HER2-targeted drugs with immunotherapy.

#### 3.2.3. Mesenchymal–Epithelial Transition (MET)

The mesenchymal–epithelial transition receptor (MET or c-MET) is one of the RTKs and is also known as a hepatocyte growth factor receptor. HGF is its sole ligand and stimulates the EMT in tumor cells and in the crosstalk with cancer-associated fibroblasts (CAFs) [111]. This interaction enhances tumor cell proliferation, survival, motility, angiogenesis, invasion, and metastasis [112]. Evidence suggests that c-MET can interact with various membrane receptors, including EGFR, FAS, IGF-IR, integrin α6β4, and CD44 [113].

Studies have shown that c-MET levels are elevated in luminal B- and HER2-overexpressing breast cancers compared to luminal A-expressing and triple-negative breast cancers [114]. High levels of phosphorylated c-MET (p-c-MET) are associated with worsened recurrence-free survival and OS in HER2+ BC. In multivariable analyses, patients with elevated c-MET levels exhibit significantly higher risks of recurrence and mortality [115]. Tumor cells with the overexpression of HER-2/neu upregulate MET receptors, contributing to trastuzumab resistance [116].

HGF binds to the ECD of c-MET, activating its intrinsic kinase activity and leading to tyrosine phosphorylation at the carboxy-terminal docking site. P-c-MET subsequently activates substrates and downstream signaling pathways, including the PI3K/AKT pathway [117]. PIK3CA and c-MET are often co-altered across various cancer types, including breast cancer. A study demonstrated that the simultaneous expression of MET and PIK3CA in an HGF-rich environment can cooperatively activate multiple oncogenic signals, including the RAS-MEK-MAPK, STAT3, and PI3K/AKT pathways. This activation promotes angiogenesis, resistance to cell death, tumor inflammation, and immune evasion, while Y-box binding protein 1 can induce progression and metastasis in various cancer types. The study also found that HGF/MET contributes to resistance to PI3K-targeted therapies, indicating the need to co-target the MET and inhibit the activation of related oncogenic pathways to mitigate resistance [118].

As an upstream regulator of E-cadherin’s N-glycosylation status, DPAGT1 can also upregulate CTHRC1 by increasing protein turnover, participating in the proliferation and migration of breast cancer cells. CAFs can promote migration and invasiveness in breast cancer cells, as well as in the EMT, by secreting CTHRC1 [119], with the PI3K pathway being one of the potential driving signaling pathways for CTHRC1 [120]. This suggests that the PI3K/AKT pathway may play a crucial role in the multiple mechanisms through which CAFs regulate the EMT.

Zanidatamab is a new drug with antitumor activity in various HER2-amplified/expressing solid tumors. In a recent phase I clinical trial, MET amplification was found to be associated with acquired resistance to the bispecific HER2-targeting antibody Zanidatamab. Additionally, it was confirmed that MET inhibitors exhibit monotherapy activity both in vitro and in vivo and can enhance the activity of Zanidatamab [121]. This provides evidence that HER2-MET crosstalk induces resistance to HER2-targeted therapies and suggests that using MET inhibitors, either alone or in combination, could be a potential therapeutic strategy.

#### 3.2.4. AXL

AXL is a transmembrane receptor belonging to the TAM (TYRO3, AXL, and MER) family. It is characterized by a kinase domain closely related to the MET and an ECD resembling that of neural cell adhesion molecules. AXL has been shown to interact with the EGFR and other members of the EGFR family [122], including HER2 and HER3 [123], thereby activating multiple signaling pathways such as PI3K, MAPK, and STAT [124]. In vitro models of lapatinib-acquired resistance HER2+ BC reveal that AXL effectively binds to the regulatory subunit p85 of PI3K, activating PI3K and circumventing the effects of lapatinib or trastuzumab. The use of inhibitors to target AXL can restore sensitivity to HER2 antagonists [125].

Moreover, AXL has been confirmed to interact with the MET [126]. GAS6 is the most common ligand of AXL, activating signaling via the involvement of the MET. MET knockout leads to reduced AXL expression. Downstream effector proteins of this interaction include AKT. Recently, it has been discovered that AXL, positioned downstream of the EMT, also promotes the EMT. This function is part of a positive feedback loop that enhances the EMT, with AXL being upregulated under the influence of EMT transcription factors and thereby increasing migration and invasion [127].

Studies on EMT in breast cancer have shown that when cells undergo a partial EMT, this can lead to lung metastasis and chemotherapy resistance, whereas fully transitioned EMT cancer cells are enriched in recurrent tumors post-chemotherapy [128]. This suggests that different stages of the EMT and the resulting plasticity of cancer cells are important mechanisms in the formation of metastatic tumors and resistance.

Mauricio Marquez-Palencia et al. discovered that the nuclear translocation of AXL interacts with WRNIP1 to mediate the DNA replication stress response. This interaction promotes resistance to HER2+ BC treatment and enhances metastatic progression. The loss or inhibition of AXL nuclear WRNIP1 prolongs the latency of metastasis and delays relapse [129]. This study suggests that AXL not only plays a critical role in the EMT process but also induces resistance to anti-HER2 therapy through other pathways.

#### 3.2.5. ER

Approximately 50% of HER2+ BC tumor cells also express ERs, and these are classified as triple-positive breast cancer (TPBC) cells. The crosstalk between HER2 and ERs can lead to resistance to both endocrine therapy and anti-HER2 treatments [130].

The ER can serve as an escape route from HER2 inhibition: ER can activate HER2 signaling through G protein interactions or by directly activating the downstream proteins of HER2. Furthermore, tumor cells that develop resistance to anti-HER2 therapies exhibit the upregulation of ER, and clinical trials have shown that TPBC demonstrates a lower response to anti-HER2 therapies compared to HER2-positive-only breast cancer cells [131]. Considering the intratumoral heterogeneity of HER2+ BC and the differences between HR+/HER2+ and HR-/HER2+ subtypes, there is an opportunity to investigate other targeted combinations in randomized clinical trials specifically aimed at HR+/HER2+ BC patients, exploring the potential for combination blockade.

In ER+ breast cancer, the levels of 14,15-EET are higher than those in non-cancerous tissues. The presence of 14,15-EET induces the EMT in breast cancer cells by activating integrin αvβ3 and the FAK/PI3K/AKT signaling cascade [132]. Concurrently, circular RNA circCDYL2 stabilizes GRB7 by preventing its ubiquitin-mediated degradation, enhancing the interaction between GRB7 and FAK. The circCDYL2-GRB7-FAK complex promotes trastuzumab resistance in breast cancer by maintaining the activity of HER2 downstream signaling pathways, including AKT and ERK1/2. Clinically, HER2+ BC patients with high levels of circCDYL2 exhibit rapid relapse after anti-HER2 therapy, as well as a shorter disease-free survival and OS compared to those with low circCDYL2 levels [133].

A promising new area of treatment for triple-positive MBC involves the incorporation of additional inhibitors targeting these signaling pathways in novel combination regimens. The combination of neratinib with fulvestrant has been shown to downregulate ER activity, pAKT, pERK, and cyclin D1 levels compared to fulvestrant monotherapy [134], underscoring the necessity of combination therapy.

The distinct molecular profiles of TPBC determine its unique clinical characteristics, including reduced chemotherapy sensitivity, different patterns of relapse, and overall favorable prognosis. A recent review summarized how the HER2-ER crosstalk is a key factor in the resistance to HER2-targeted drugs. Endocrine therapy has shown good efficacy. As dual-target drugs for both ER and HER2, PAM pathway inhibitors, including CDK4/6 inhibitors, can address the resistance mechanisms of both, potentially further enhancing efficacy [135]. These data preliminarily suggest that combination therapy involving antibody–drug conjugates better aligns with the unique biological characteristics of TPBC tumors.

#### 3.2.6. Other Pathways

The ubiquitination of the AKT is an important step in the activation of the oncogenic AKT [136], with HER2 and other members of the ERBB family playing regulatory roles in this process. Skp2-SCF E3 ligase is a key E3 ligase involved in ERBB receptor-mediated AKT ubiquitination and membrane recruitment. Skp2 can either induce proteasome-mediated proteolysis or regulate the function of substrate-tagged proteins [137]. In various tumor models, the absence of Skp2 impairs AKT activation, Glut1 expression, glucose uptake, glycolysis, and the progression of breast cancer. The overexpression of Skp2 is associated with AKT activation and breast cancer metastasis and may serve as a prognostic marker of poor outcomes in HER2+ patients. Silencing Skp2 can sensitize HER2-overexpressing tumors to trastuzumab treatment [138].

Research has shown that the AKT directly induces the phosphorylation of senescence-like tetraspanin-8 (TSPAN8) at Ser129, promoting its interaction with 14-3-3θ/importin-β and its subsequent nuclear transport. Once translocated to the nucleus, TSPAN8 interacts with STAT3, thereby regulating chromatin remodeling and influencing the transcription of downstream oncogenes such as MYC, BCL2, and MMP9. This reveals a potential mechanism by which the EGFR-AKT-TSPAN8-STAT3 axis negatively affects prognosis and treatment resistance in breast cancer and other malignancies [139]. Recently, a subpopulation of TSPAN8+ myofibroblast-associated fibroblasts (myCAFs) has been identified as being associated with treatment resistance and poor survival rates in multiple breast cancer patient cohorts [140]. The mechanisms by which CAFs contribute to chemotherapy resistance in breast cancer remain unclear, but this study provides new insights. The PI3K pathway has multiple connections with the functions of CAFs, potentially playing a role in this process.

In HER2+ BC, the PI3K/AKT pathway forms a highly coordinated signaling network through its close interactions with multiple other signaling pathways. On the one hand, the interaction between HER2 and HER3, along with crosstalk with the ER, accelerates cell division and enhances tumor cell resistance to apoptotic signals. This complex interplay promotes tumor cell survival and proliferation, exacerbating resistance to anti-HER2 therapies.

On the other hand, this intricate signaling interaction extends beyond the tumor cells themselves. It also intensifies resistance through intercellular communication within the tumor microenvironment. The activation of the PI3K/AKT pathway mobilizes TAMs and CAFs, which promote the release of immunosuppressive cytokines and pro-invasive factors. These factors interact with receptors on the tumor cell surface, enhancing PI3K/AKT signaling and directly regulating the expression of EMT-related genes. This regulation drives tumor cells to adopt a more invasive phenotype. Additionally, the cytoskeletal reorganization and decreased cell adhesion associated with the EMT facilitate the penetration of tumor cells through the basement membrane into the bloodstream, promoting metastasis.

Ultimately, these interactions contribute to the failure of anti-HER2 targeted therapies. Understanding these multi-pathway interactions provides a critical foundation for developing new combination treatment strategies. Specifically, the potential for dual blockade targeting these intersecting signals may offer new hope for overcoming resistance.

## 4. Clinical Applications of the PI3K/AKT Pathway in Targeted Therapy Resistance in HER2-Positive Breast Cancer

Due to the complexity of the HER2 network, an increasing number of studies are focusing on combination therapies that utilize HER2-targeted drugs alongside various other agents, such as PI3K inhibitors and angiogenesis inhibitors. Although research in this area is still evolving, preclinical data suggest that combining specific PI3K inhibitors with existing anti-HER2 therapies may enhance their efficacy and help to avoid resistance in cases of PIK3CA-mutant HER2+ advanced breast cancer. Consequently, testing for PIK3CA mutations before initiating anti-HER2 treatment may soon become a necessary condition in terms of ensuring durable and effective responses in this indication.

There are now kits available for monitoring PIK3CA mutations, and additional scoring methods for assessing PI3K pathway activity are under development [141], enabling timely adjustments in therapy that may enable us to improve patient outcomes [33]. The FDA has approved the use of therascreen in tumor tissue and plasma and FoundationOne CDx in tissue samples for the detection of PIK3CA mutations in breast cancer patients. In HER2+ BC patients with PIK3CA mutations, combination therapies utilizing HER2 inhibitors and next-generation PI3K pathway inhibitors, such as AKT inhibitors, as well as other PI3K pathway inhibitors can overcome the therapeutic limitations of monotherapy with anti-HER2 agents in PIK3CA-mutant HER2+ BC cell lines [24]. Dual blockade treatment for HER2+ BC not only enhances the therapeutic efficacy but also reduces the need for unnecessary treatment, thereby alleviating associated toxicities and patient costs (Table 2).

### 4.1. PI3K Inhibitors

Preclinical studies indicate that HER2 signaling is predominantly mediated through p110α rather than any of the other three catalytic subunits of PI3K, suggesting that PI3K inhibitors, especially selective PI3K inhibitors, have the potential to overcome resistance to anti-HER2 therapies [176].

The first study was focused on pan-PI3K inhibitors, with representative drugs including Buparlisib (BKM120), Pilaralisib (XL147), and Pictilisib (GDC-0941).

Buparlisib (BKM120) is an effective pan-PI3K inhibitor [177]. The PIKHER2 trial demonstrated a clinical benefit rate (CBR) of 29% when Buparlisib was combined with lapatinib in patients with advanced HER2+ BC who were resistant to trastuzumab and had PIK3CA mutations, with one patient (4%) achieving a complete response (CR), indicating the feasibility of this combination regimen [143]. Another phase Ib/II trial evaluated the effects of Buparlisib combined with trastuzumab in trastuzumab-resistant tumors in patients with advanced HER2+ BC, observing evidence of clinical activity (2% CR and 8% partial responses) [144]. A small phase I trial investigated the combination of capecitabine and Buparlisib in HER2+ MBC patients, which resulted in a complete response rate of 4% and a partial response rate of 16% compared to the placebo group [145]. However, in the NeoPHOEBE phase II trial, HER2+ eBC patients were randomly assigned to receive either Buparlisib or a placebo in combination with paclitaxel and trastuzumab. This treatment group did not demonstrate significant benefits, with a pCR rate of 32% in the Buparlisib group compared to 40% in the placebo group [142].

Copanlisib (BAY 80-6946) is another potent pan-PI3K inhibitor that was approved by the FDA in May 2017 for the treatment of relapsed refractory follicular lymphoma [178]. Its use has also been investigated in breast cancer, particularly in HER2+ BC. The Panther and Panthera trials evaluated the efficacy of Copanlisib in combination with trastuzumab or T-DM1 in patients with HER2+ MBC and advanced breast cancer (ABC). Although all participants discontinued treatment due to disease progression, differences in PIK3CA variant allele frequency were observed between pre- and post-treatment tumors, indicating that Copanlisib may exert inhibitory effects on clones with PIK3CA mutations, warranting further exploration [147].

Other pan-PI3K inhibitors, such as Pictilisib (GDC-0941) [179] and Pilaralisib (XL147) [146], have been studied in multiple clinical trials, including both HER2+ and HER2- breast cancer cohorts. However, most data relate to ER+/HER2- breast cancer, and while there has been research on HER2+ patients in subgroup analyses, current clinical studies have not demonstrated substantial efficacy, necessitating further investigation.

The FDA has approved the combination of pan-PI3K inhibitors with fulvestrant for ER+ BC; however, preclinical studies have demonstrated toxicity when targeting the PI3K pathway in conjunction with anti-HER2 inhibitors. Pan-PI3K inhibitors have been shown to simultaneously inhibit glucose uptake and disrupt insulin metabolism, leading to dose-limiting hyperglycemia and hyperinsulinemia [180]. This has resulted in no pan-PI3K inhibitor being successfully approved for use in the field of breast cancer to date. Therefore, there is a pressing need for the development of more effective and safer selective PI3K inhibitors.

As the understanding of the protein structure and isoform specificity has deepened, isoform-specific PI3K inhibitors have officially emerged in the field of anticancer therapy. These inhibitors can be classified into α, β, δ, and γ isoforms based on the different catalytic subunits they target. PI3Kα inhibitors, including Alpelisib (BYL719), Taselisib (GDC-0032), and Inavolisib (GDC-0077), show notable efficacy in breast cancer treatment. Several clinical studies have demonstrated that PI3Kα inhibitors successfully address safety concerns.

Alpelisib (BYL719) is a selective inhibitor of the PI3K α isoform and has been extensively studied in breast cancer patients with PIK3CA mutations [181]. A phase I trial investigated the combination of alpelisib and T-DM1 in HER2+ MBC patients who were resistant to trastuzumab and paclitaxel. In this study, the objective response rate (ORR) was 43%, and in patients whose disease had worsened after previous T-DM1 treatment (N = 10), the ORR was 30% with a CBR of 60%. Among the 17 patients enrolled, 9 (53%) exhibited molecular alterations in the PI3K pathway (including PTEN loss, AKT overexpression, or PIK3CA mutations), with 5 of these patients (56%) achieving a CBR, including 3 who progressed during T-DM1 therapy [148]. Further studies on the use of alpelisib to treat HER2+ BC are ongoing, including the EPIK-B2 trial, which is assessing the combination of alpelisib with trastuzumab and pertuzumab as maintenance therapy in patients with HER2+ ABC harboring PIK3CA mutations [150]. The ALPHABET trial is a randomized phase III study evaluating the efficacy of alpelisib plus trastuzumab with or without fulvestrant in previously treated HER2+ patients with PIK3CA mutations. This is compared to the use trastuzumab combined with physician-selected chemotherapy (eribulin, capecitabine, or vinorelbine) [151].

Taselisib (GDC-0032) is another selective and effective inhibitor targeting class I PI3K subunits p110α, p110γ, and p110δ, with preferential activity against mutant forms of p110α. In the context of ER+/HER2- MBC, the SANDPIPER study evaluated the efficacy of taselisib in combination with fulvestrant. Although the inclusion of taselisib resulted in a statistically significant improvement in PFS, the drug was deemed unfavorable for further development due to its moderate clinical activity and associated toxicity [182]. A recent phase I trial assessed the effects of taselisib combined with T-DM1, demonstrating significant benefits for patients who had previously progressed when treated with T-DM1 [152].

Inavolisib (GDC-0077) is a novel PI3Kα inhibitor [183] and offers high selectivity and specificity, effectively inhibiting tumor growth driven by PIK3CA mutations while minimizing the inhibition of wild-type PI3K, thereby reducing side effects. In the ER+ breast cancer population, inavolisib, either as a monotherapy or in combination therapy, has demonstrated good safety in both the overall population and long-term treatment groups, with manageable overall safety profiles [184]. Recently, the FDA approved the use of inavolisib with palbociclib and fulvestrant for endocrine-resistant, PIK3CA-mutated HR+ and HER2- advanced breast cancer. This highlights its significant potential and provides great encouragement for future therapeutic advancements. Clinical studies of the use of inavolisib in HER2+ breast cancer, particularly in patients with PIK3CA mutations, have gradually been initiated over the past two years [153,154,185].

Interestingly, certain drugs share common structural features. For instance, both taselisib and inavolisib contain a condensed heterocyclic system with three rings. However, such a structure is absent in alpelisib, which instead possesses an amide group on its five-membered ring, similar to that found in taselisib. This suggests that these common molecular pharmacophores may play a critical role in exerting PI3K-inhibitory effects. These findings highlight the importance of focusing on such shared molecular pharmacological features in future research.

New PI3K inhibitors are also currently under development. A novel proteolysis-targeting chimera (PROTAC) has been developed that specifically targets PI3K-p110α. This effectively inhibits the proliferation of breast cancer cells by degrading PI3K-p110α and inducing G1 phase cell cycle arrest. This approach has also been shown to restore sensitivity to lapatinib, exhibiting greater efficacy than the use of alpelisib [186]. In ER+ BC, the selective PI3K inhibitor RLY-2608 targets allosteric sites on PI3K, offering better efficacy than others in this regard, and is now the focus of clinical research [187]. This provides additional possibilities for overcoming resistance to anti-HER2 therapies in HER2+ BC patients.

Currently, research on PI3K inhibitors in HER2+ BC has primarily focused on patients with PIK3CA mutations. Although some clinical trials have been conducted within the HER2+ subgroup, much of the research has concentrated on HER2- subtypes, particularly ER+/HER2- breast cancer. In the future, more clinical trials are anticipated to specifically target HER2+ BC patients.

### 4.2. AKT Inhibitors

Currently, targeted drugs aimed at the PAM signaling pathway primarily consist of PI3K inhibitors and mTOR inhibitors, with the AKT positioned at the core of this signaling cascade. Inhibiting AKT activity can avoid the severe side effects associated with upstream PI3K inhibition and mitigate the impact of negative feedback mechanisms resulting from downstream mTOR inhibition, making this an important direction in cancer drug development [188]. However, the development of AKT inhibitors has not been straightforward. Major multinational corporations such as AstraZeneca, Roche, and Merck have all experienced lengthy and challenging drug development processes in this field. It was not until November 2023 that the first AKT inhibitor, Capivasertib, developed by AstraZeneca, received FDA approval, confirming the druggability of the AKT target.

There are various methods for inhibiting AKT activity, and AKT inhibitors are categorized as ATP-competitive inhibitors, allosteric inhibitors, or irreversible inhibitors. Classic ATP-competitive inhibitors were developed to prevent the AKT from phosphorylating its substrates. Studies have validated that the concurrent use of ATP-competitive inhibitors to target the AKT/mTOR pathway and HER2 inhibitors can induce apoptosis in tumor cells and result in tumor shrinkage, with these effects being sustained over time. Recently, several companies have disclosed compounds with oral bioavailability, with a few currently undergoing phase I clinical trials. The first compound described in detail was GSK690693, which, when used in conjunction with lapatinib, can attenuate the compensatory upregulation of p-HER3 and p-HER2 and thereby offset the effects of PIK3CA mutations [189]. Although this compound demonstrates strong efficacy and specificity, it lacks oral bioavailability, leading to its withdrawal from development during phase I trials.

Capivasertib (AZD5363) is a selective ATP-competitive AKT inhibitor that targets AKT1, AKT2, and AKT3, demonstrating potential for use in various cancers, including HER2+ BC. In breast cancer models, Capivasertib has shown the ability to inhibit the growth of a range of human tumor xenografts, both as a monotherapy and in combination with HER2 inhibitors. Notably, the combination of Capivasertib with docetaxel in breast cancer xenografts resulted in significantly pronounced tumor regression. Based on these promising preclinical findings, Capivasertib is currently undergoing phase I clinical trials [190]. The FAKTION trial is a phase II study investigating the efficacy of Capivasertib in combination with fulvestrant for patients with ER+ and PIK3CA-mutant breast cancer. In HER2+ BC, Capivasertib has also exhibited notable activity, particularly in patients harboring mutations in the PI3K/AKT pathway [191].

Ipatasertib (GDC-0068) is another selective ATP-competitive inhibitor that targets AKT1, AKT2, and AKT3. The combination of Ipatasertib with lapatinib has successfully overcome resistance to HER2 treatment in vitro in PIK3CA-mutant HER2+ breast cancer cells. In preclinical models, this compound has demonstrated the ability to enhance the antitumor efficacy of both chemotherapy agents and molecular-targeted therapies, and it is currently in phase II clinical trials [155]. The IPATHER study is a phase Ib trial designed to evaluate the safety and efficacy of Ipatasertib in combination with trastuzumab and pertuzumab in patients with locally advanced/unresectable or metastatic PIK3CA-mutant HER2+ breast cancer. Early results indicate that participants tolerate Ipatasertib well, and preliminary signs of efficacy have been observed [156].

The high homology of AKT isoforms has made the development of selective AKT inhibitors particularly challenging. As a result, researchers have gradually shifted their focus towards allosteric inhibitors of AKT, aiming to achieve greater isoform selectivity and specificity.

MK-2206 is a potent and highly specific non-ATP-competitive allosteric inhibitor of AKT that binds to regions interacting with the AKT homology and kinase domains. This binding prevents AKT translocation to the membrane and its subsequent activation, thereby inhibiting tumor growth. Clinical phases I/II trials have evaluated the combination of MK-2206 with chemotherapeutic agents in HER2+ BC patients, showing promise treating those patients resistant to HER2-targeted therapies [158,159,160,161]. Additional studies have explored the efficacy of combining MK-2206 with trastuzumab, with results indicating a favorable resistance profile and a CBR (CR, partial response, and stable disease for ≥4 months) of 24% [162]. In the I-SPY2 trial, the pan-AKT inhibitor MK-2206 was tested in combination with neoadjuvant trastuzumab and paclitaxel, resulting in an increase in the pCR rate from 29% to 48%.

Research on the use of other AKT inhibitors, such as perifosine [163], afuresertib (GSK2110183), and uprosertib (GSK2141795), in HER2+ BC is limited, although some early studies have explored their potential in combination with HER2-targeted therapies. Once again, similar structures have been identified in ipatasertib and capivasertib. This indicates that these structural elements may be crucial for the function of AKT inhibitors, reiterating the importance of focusing on molecular pharmacological characteristics.

AKT inhibitors have shown clinical potential in HER2+ BC, particularly in patients with PIK3CA mutations or PTEN loss. However, most studies are still in phase I/II, with many focusing on HER2- breast cancer or other types of cancer. The response to AKT inhibitors in HER2+ BC is still under investigation and undergoing validation, and future clinical trials may provide more data on their effectiveness.

### 4.3. mTOR Inhibitors

mTOR inhibitors are primarily categorized into three classes: antibiotic allosteric mTOR inhibitors (first generation), ATP-competitive mTOR inhibitors (second generation), and other novel mTOR inhibitors (third generation) [192]. Most mTOR inhibitors undergoing clinical research belong to the first generation.

Everolimus (RAD001) is an mTORC1 inhibitor that reduces cancer cell proliferation and induces apoptosis by inhibiting mTOR, thereby reversing trastuzumab resistance caused by the overactivation of the PAM pathway due to PTEN loss. The application of Everolimus in breast cancer has been extensively studied in HR+ BC, and research is gradually progressing in HER2+ BC, particularly in patients resistant to HER2-targeted therapies such as trastuzumab [174]. A phase II clinical trial focusing on the use of Everolimus in MBC primarily targeted HR+ BC and showed some clinical benefit; however, the HER2+ subgroup did not demonstrate a significant response [164]. In contrast, results from a phase I trial involving patients with metastatic HER2-overexpressing breast cancer indicated that patients generally tolerated the combination of Everolimus with trastuzumab and paclitaxel well, showing encouraging antitumor activity. In particular, we saw an ORR of 55% in trastuzumab-resistant patients [165]. The combination of Everolimus with trastuzumab and vinorelbine also exhibited some effectiveness, with an ORR of 19.1% and a disease control rate of 83.0% [166].

The BOLERO series of trials demonstrated potential for improving PFS. The BOLERO-1 trial evaluated the safety and efficacy of adding Everolimus to trastuzumab and paclitaxel as the first-line treatment for advanced HER2+ BC patients. The combination of Everolimus with trastuzumab and taxanes resulted in significant clinical responses in metastatic HER2+ BC patients who had previously experienced disease progression when treated with trastuzumab, extending the PFS by 7–12 months [168]. The BOLERO-3 trial assessed the combination of Everolimus with trastuzumab and chemotherapy (vinorelbine) in HER2+ patients with MBC resistant to trastuzumab. The results showed that Everolimus combination therapy significantly improved the PFS compared to the use of trastuzumab plus chemotherapy alone [171]. Moreover, patients with a poor prognosis due to brain metastases from breast cancer benefited from treatments combining Everolimus with trastuzumab and vinorelbine or with lapatinib and capecitabine. In a phase II trial, one-third of patients achieved a CNS CBR [172], and in another trial, the CNS response rate was 27% at 12 weeks [173]. However, in the large, phase III GeparQuinto trial, the chemotherapy regimen combining paclitaxel with Everolimus in early HER2+ BC demonstrated good safety but did not lead to significant clinical benefits [167].

Temsirolimus (CCI-779) is an intravenous mTOR inhibitor. Clinical trials of Temsirolimus in combination with neratinib have been conducted in HER2+ BC patients, primarily focusing on its efficacy in those resistant to HER2-targeted therapies. These have shown some clinical activity. Ridaforolimus (AP23573, MK-8669), also known as deforolimus, is another derivative of rapamycin. The phase II trial 8669-009 evaluated the efficacy of ridaforolimus in combination with trastuzumab in a population of HER2+ MBC patients who were resistant to trastuzumab, demonstrating significant antitumor activity (CBR, 34.3%; PFS, 5.4 months; OS, 17.7 months) [175].

Dactolisib (BEZ235) is a dual inhibitor of PI3K and mTOR that can downregulate the AKT-mTOR-HIF1α signaling pathway, inhibiting tumor-induced angiogenesis and promoting cell death through apoptotic pathways. This compound significantly suppresses tumor growth in models of trastuzumab-resistant HER2+ BC [193]. The NCT0147184 trial aims to assess the safety and efficacy of BEZ235 in combination with trastuzumab for trastuzumab-resistant HER2+ breast cancer patients, comparing it to the use of lapatinib plus capecitabine in the same patient cohort.

In addition to these major drugs, other mTOR inhibitors, such as rapamycin and apitolisib (GDC-0980), have also been preliminarily evaluated in the context of HER2+ BC. Additionally, the third-generation mTOR inhibitor Rapalink-1 combines rapamycin and the mTOR kinase inhibitor MLN018 into a single molecule, simultaneously targeting mTOR and its kinase domain [194]. This dual inhibition has demonstrated potent antitumor effects in preclinical models of breast cancer and various other cancers [195]. Future research may further investigate combinations of mTOR inhibitors with HER2-targeted therapies and their application across different resistance mechanisms.

### 4.4. Other Pathway Inhibitors

Considering the role of HER3 in anti-HER2 resistance, the combination of anti-HER2 and HER3-targeting drugs with PI3K pathway inhibitors has emerged as a new approach. A phase I trial investigated the maximum tolerated dose (MTD) of alpelisib in combination with trastuzumab and the HER3-targeting antibody LJM716 for patients with PIK3CA-mutant HER2+ MBC. Among the 17 evaluated patients, 1 experienced a partial response, 14 exhibited stable disease, and 2 showed a full response. This indicates that the combination therapy of alpelisib, trastuzumab, and LJM716 is limited by gastrointestinal toxicity. Further efforts are needed regarding the PI3K pathway in HER2+ MBC [149]. Additionally, the newly developed HER3-targeting drug SAR256212 is also being studied in combination with PI3K inhibitors [196].

The results of these clinical trials demonstrate that PI3K/AKT inhibitors, either as monotherapy or in combination regimens, exhibit manageable drug-related adverse effects. Moreover, the maximum tolerated doses of these agents have been established, providing a foundation for further clinical investigation. In several trials, the combination of PI3K/AKT-targeted therapies with HER2-targeted agents improved outcomes such as pCR, CBR, and PFS in HER2+ BC patients.

These findings suggest that HER2+ BC patients, particularly those with advanced disease and resistance to HER2-targeted therapies, may benefit from the addition of PI3K/AKT inhibitors during neoadjuvant or adjuvant treatment phases. This approach appears to enhance the therapeutic efficacy of HER2-targeted regimens. The evidence from these trials offers promising new strategies for the management of HER2+ BC patients who develop resistance to existing therapies.

## 5. Future and Perspective

### 5.1. Impact of Different Populations on the Efficacy of PI3K/AKT-Targeted Therapy in HER+ BC

Although PI3K/AKT-targeted therapies have expanded the treatment options for breast cancer, especially for metastatic breast cancer, there are still disparities in efficacy across different regions and ethnic groups. Real-world studies have shown that the use of PI3K pathway inhibitors is also influenced by socio-economic factors.

Compared to white women, black women often have worse breast cancer-related prognoses and higher mortality rates. While there is no significant difference in the efficacy of PI3K/mTOR inhibitors between black/African American patients and white patients, the incidence of drug-induced hyperglycemia is twice as high in black patients (OR 2.02, CI 1.24–3.39, *p* < 0.01) [197]. On the other hand, racial differences also contribute to an increased cardiovascular toxicity burden related to cancer treatment. Black women are at twice the risk of cardiovascular toxicity (HR, 2.73; CI, 1.24–6.01). Cardiovascular toxicity is an adverse effect that must be closely monitored with PI3K inhibitors, as PI3K pathway inhibition may lead to cardiac dysfunction, electrical remodeling, and vascular damage [198].

However, some studies indicate that there are no differences in the final approved doses of single-agent targeted therapies, including PI3K/mTOR inhibitors (e.g., everolimus), between North America and Asia. Despite differences in race, body weight, and BMI, there are no differences in the recommended phase 2 doses, toxicity, pharmacokinetics, pharmacodynamics, or efficacy [199]. It may be unnecessary to conduct separate monotherapy clinical trials in white and Asian populations, but we must still consider socio-economic and cultural factors. More efforts are required to ensure the fairness and tolerability of new therapies across different populations. Additionally, given the genomic differences between black, white, and Asian populations, it is essential to determine more precise drug dose and toxicity assessments tailored to different racial groups. Drug development strategies should fully consider population-specific factors that may affect drug metabolism, safety, and efficacy, especially in HER2+ BC, where PI3K/AKT is often involved in combination with various targeted or chemotherapy drugs.

### 5.2. Potential Biomarkers of the PI3K/AKT Pathway

The role of the PI3K/AKT pathway in resistance to anti-HER2 therapies in HER2+ breast cancer (HER2+ BC) has been discussed previously. Treatment decisions for PI3K/AKT-targeted therapies are influenced by mutations in PI3K/AKT/PTEN. Therefore, identifying predictive biomarkers to assist clinicians in selecting the most appropriate and personalized treatment for HER2+ BC is crucial. Modern clinical trials commonly use PIK3CA mutations, PTEN loss, and AKT mutations as prognostic and predictive markers [200]. Additionally, pAKT and p-mTOR, which indicate the activation of the PAM pathway, are frequently used. Other potential markers include TRRAP, angiogenesis markers (VEGF, VEGFC, VEGFD, sVEGFR1, sVEGFR2, bFGF, cKIT, TIE2, PIGF), and cell death markers (M30, M65).

A phase I/II clinical trial demonstrated that patients with PIK3CA mutations in tumor or circulating DNA responded well to treatment with Alpelisib in combination with albumin-bound paclitaxel in HER2-negative breast cancer. However, metabolic differences due to diabetes were found to influence treatment efficacy [201]. For HER2+ BC, one study summarized the results of four prospective clinical trials and found that the PIK3CA hotspot mutation p.H1047R was associated with a decreased pathologic complete response (pCR) rate in HER2-positive breast cancer patients undergoing neoadjuvant therapy, while hotspot mutations in exon 9 appeared to have a lesser effect [202]. PIK3CA mutations are also linked to specific gene expression profiles, such as the upregulation of angiogenesis markers and downregulation of MYC. In other cancers, such as cervical and prostate cancer, PIK3CA mutations have been associated with altered subcellular localization of FOXO3a [203] and lower levels of expression of immune-related genes [204], making them potential biomarkers. These findings provide valuable insights for the study of PI3K/AKT pathway-related biomarkers in HER2+ BC.

To optimize the efficacy of PI3K/AKT-targeted therapies, it is crucial to accurately assess alterations in the PI3K/AKT pathway in HER2+ BC patients to fully exploit the potential benefits of these therapies. In addition to comprehensive genetic testing, diagnostic kits for detecting individual mutations in the PI3K/AKT pathway and its components (such as PIK3CA mutations and PTEN loss) have been developed. In 2019, following the FDA approval of the PI3K inhibitor Alpelisib, the QIAGEN therascreen PIK3CA RGQ PCR kit was also approved as a companion diagnostic product for detecting PIK3CA mutations in tissue and/or peripheral blood ctDNA (liquid biopsy). Subsequently, the breast cancer guidelines issued by the NCCN, ASCO, and ESMO incorporated PIK3CA gene mutation testing into clinical pathways. However, it is important to note that these approvals and guidelines currently focus on the diagnosis and treatment of HR+/HER2- breast cancer. For HER2+ BC, further research is needed to explore the impact of PI3K/AKT pathway alterations on targeted therapies. It is also hoped that the detection of PI3K/AKT pathway alterations will eventually be included in the recommended clinical pathways for HER2+ BC in future breast cancer guidelines.

### 5.3. Safety of PI3K/AKT-Targeted Therapies

The safety of drugs has always been a key focus during the development of new medications and therapies. The FDA has approved several PI3K/AKT pathway inhibitors for the treatment of breast and other cancers, including PI3K inhibitors (Copanlisib and Alpelisib), AKT inhibitors (Capivasertib), and mTOR inhibitors (Sirolimus, Everolimus, and Temsirolimus). These approvals demonstrate that PI3K/AKT-targeted therapies have been shown to be safe under specified usage conditions after rigorous clinical trials. Moreover, the side effects and adverse reactions observed in these trials were within acceptable limits.

As previously mentioned, several clinical trials are currently investigating HER2 and PI3K/AKT combined targeted therapies for HER2+ breast cancer (HER2+ BC). Within these phase I or II clinical trials, the safety and dosing of certain PI3K inhibitors, AKT inhibitors, and mTOR inhibitors have been effectively assessed [143,146,148,152,158,164,168,175]. However, some studies suggest that these targeted therapies may cause grade 3 or higher treatment-related adverse events that could affect drug tolerability, causing liver toxicity [142] or adverse gastrointestinal effects [149]. In some trials, these side effects were considered acceptable. Ongoing clinical trials will provide further insights into the safety of PI3K/AKT-targeted therapies in HER2+ BC.

Therefore, we can conclude that the safety of PI3K inhibitors, AKT inhibitors, and mTOR inhibitors in clinical use is acceptable, but continuous monitoring and management during treatment remain necessary.

### 5.4. Regulatory Environment and Ethical Considerations in the Use of PI3K/AKT-Targeted Therapies

The primary regulatory concern for the use of PI3K/AKT-targeted therapies in HER2+ breast cancer (HER2+ BC) is the assessment of treatment efficacy. On one hand, it is essential to regularly evaluate patient responses to treatment, particularly in patients who have developed resistance to anti-HER2 therapies. Monitoring tumor shrinkage and assessing whether the disease is stable or progressing are crucial for timely adjustments to the treatment regimen. On the other hand, testing for PI3K/AKT pathway biomarkers is necessary to evaluate the effectiveness of the treatment. Additionally, monitoring drug safety is critical. Regular testing of liver function, blood glucose levels, and other relevant indicators, as well as monitoring for comorbidities, is vital. In addition to addressing common treatment-related adverse effects, this helps to identify potential side effects. Moreover, considering that neoadjuvant or adjuvant therapies for HER2+ BC often involve combination therapies, monitoring drug interactions is necessary to adjust treatment plans and ensure the safety of medication use.

The application of PI3K/AKT-targeted therapies in HER2+ BC is still in the clinical trial phase. Therefore, ethical review is a crucial issue. First, patient-informed consent must be ensured, with complete information provided and adequate understanding ensured to allow patients to make informed decisions regarding their treatment. Second, the fairness of resource allocation in patient selection needs to be considered to avoid disparities based on race, gender, economic status, and other factors. Additionally, ethical committees must continue to oversee the PI3K/AKT-targeted treatment process, adjusting treatment plans as needed to address emerging ethical issues.

Finally, strict project management, ethical review, and quality control are essential requirements for the application of PI3K/AKT-targeted drugs in HER2+ BC treatment. Regulatory efforts for PI3K/AKT-targeted therapies require long-term commitment, not only in the evaluation of immediate efficacy but also in the assessment and follow-up of long-term impacts. Psychological support and disease education for patients are also critical components of the treatment process.

### 5.5. Challenges of PI3K/AKT-Targeted Therapy from Preclinical to Clinical Application

One of the major challenges in the development and clinical application of PI3K/AKT-targeted drugs is drug accessibility and market acceptance. This includes the determination of clinical efficacy and economic evaluation. For PI3K/AKT-targeted therapies in HER2+ breast cancer (HER2+ BC), it is necessary to incorporate PI3K/AKT pathway biomarker testing alongside the conventional diagnostic criteria for HER2+ BC. FISH testing for HER2 has become an essential part of molecular profiling in breast cancer diagnosis. This standardization underwent a lengthy clinical trial process supported by extensive clinical and basic research data, demonstrating the critical role of HER2 assessment in treatment and prognosis. In contrast, for the PI3K/AKT pathway, the development of biomarker testing kits is still ongoing, and currently there are no guidelines that include testing PI3K/AKT pathway mutations in diagnostic standards. Moreover, the high costs associated with genetic testing are likely to hinder the widespread application of PI3K/AKT-targeted therapy among HER2+ BC patients.

Additionally, the approval process for new drugs and treatments in various countries is complex, requiring adherence to strict standards and guidelines. Clinical trials for PI3K/AKT-targeted drugs are still ongoing. While some drugs have already been approved, their indications in HER2+ BC remain limited, necessitating further time and financial investment to support the development and approval of PI3K/AKT-targeted therapies. The investment risks involved pose a challenge for startups and research institutions.

Overall, although there are many challenges in moving PI3K/AKT-targeted therapies from the preclinical stage to clinical application, we remain confident in their potential. More research is also needed to explore the application of PI3K/AKT-targeted therapy in HER2+ BC and to validate its potential in overcoming resistance to anti-HER2 treatments.

## 6. Conclusions

In summary, resistance to HER2-targeted therapies remains a significant challenge in the treatment of HER2+ BC. The PI3K/AKT pathway plays a critical role in the development of resistance to targeted therapies in HER2+ BC. Various mechanisms contribute to this resistance, including alterations in the downstream pathways of PI3K/AKT signaling and their interactions with other pathways. Strategies that combine anti-HER2 drugs with inhibitors targeting members of the PI3K pathway have shown potential clinical value in preclinical models and clinical trials.

## Figures and Tables

**Table 1:** Frequency of PI3K/AKT alterations in HER2+ breast cancer.

Element	Type of Alteration	Frequency	Dataset
PI3K	PIK3CA mutation	38.6%	TCGA [25]
		29.5% (ER+), 30.1% (ER-)	METABRICK [26]
		31%	MSK [27]
		32%	NeoSphere [28]
		31.6%	CLEOPATRA [29]
	PIK3CA amplification	30%	TCGA
	PIK3R1 mutation	7%	TCGA
	PIK3R1 amplification	1.8%	TCGA
	PIK3R3 amplification	3.6%	TCGA
AKT	AKT1 mutation	1.8%	TCGA
		2.44%	CBCGA [30]
	AKT1 amplification	20%	TCGA
	AKT2 amplification	10%	TCGA
	AKT3 amplification	67%	TCGA
	Increase in AKT protein levels	Cytoplasmic 57%, Nuclear 50%	CLEOPATRA
PTEN	PTEN mutation	1.8%	TCGA
		3.25%	CBCGA
	PTEN loss	16.4%	TCGA
		30.7%	CBCGA
	Reduction in PTEN protein levels	Cytoplasmic 48.1%, Nuclear 47.4%	CLEOPATRA
INPP4B	INPP4B loss	29.1%	TCGA

**Abbreviations**: PI3K, phosphoinositide 3-kinase; PTEN, phosphatase and tension homolog; ER, estrogen receptor; INPP4B, inositol polyphosphate-4-phosphate type II B.

**Table 2:** Ongoing clinical trials for new drugs to overcome resistance to anti-HER2 agents.

Type of Targeted Agents	PI3K/AKT Inhibitor	Study Design	Identifier	Cancer Type	Combination Therapy	Sample Size (n)	Statistical Analysis	Results	Reference
PI3K inhibitor									
pan-PI3Ki									
	Buparlisib (BKM120)	Phase II, RCT	NCT01816594, NeoPHOEBE	HER2+, PIK3CA+	Trastuzumab, paclitaxel	50	pCR, ORR (Fisher exact test, Clopper and Pearson method)	ER+ subgroup benefits	[142]
		Phase Ib, SAT	NCT01589861, PIKHER2	HER2+, PIK3CA+, trastuzumab-resistant	Lapatinib	106	dose-escalation design (BLRM), DLT, MTD, RP2D, PK	MTD (Buparlisib 80 mg/d + Lapatinib 1250 mg/d), CBR 29%	[143]
		Phase Ib/II, SAT	NCT01132664	HER2+, ABC	Trastuzumab	72	ORR (BLRM), PFS (Kaplan–Meier analysis)	CR 2%, PR 8%, ORR 10%	[144]
		Phase I, SAT	NCT01300962	HER2+, MBC	Capecitabine, trastuzumab	47	DLT, MTD, PK	CR 4%, PR 16%	[145]
	Pilaralisib (XL147)	Phase II, non-RCT	NCT01042925	HER2+, MBC	Trastuzumab, paclitaxel	42	DLT, MTD, ORR, PFS, PK	MTD (400 mg once daily), PR 20%	[146]
	Pictilisib (GDC-0941)	Phase Ib, non-RCT	NCT00928330	HER2+, MBC	T-DM1, trastuzumab	57	NA	No result published	
	Copanlisib (BAY 80-6946)	Phase Ib/II, RCT	NCT04108858	HER2+, ABC	Trastuzumab, Pertuzumab	2	RP2D	Terminated	
		Phase I, SAT	NCT04042051, Panthera	HER2+, ABC/MBC	T-DM1	2	NA	Terminated	
		Phase I, SAT	NCT02705859, Panther	HER2+, ABC/MBC	Trastuzumab	26	DLT, MTD	MTD (60 mg plus trastuzumab 2 mg/kg weekly)	[147]
Selective PI3K inhibitor									
	Alpelisib (BYL719)	Retrospective Observational Study	NCT05982886	HER2−/+, HR−/+, PIK3CA−/+		1093	NA	No result published	
		Phase Ib/II, SAT	NCT05230810	HER2+, PIK3CA+, MBC	Tucatinib	40	DLT, MTD, PFS, ORR, CBR, DOR	No result published	
		Phase I, SAT	NCT01300962	HER2+, MBC	Capecitabine, trastuzumab	47	DLT, MTD	No result published	
		Phase I, SAT	NCT02038010	HER2+, MBC	T-DM1	17	DLT, MTD, ORR, CBR, PFS (Kaplan–Meier curves)	ORR 43%, CBR 71%, PFS 8.1 months	[148]
		Phase I, SAT	NCT02167854	HER2+, MBC	LJM716, trastuzumab	23	DLT (CRM), MTD, PFS, CBR	MTD (350 mg), CBR 88%	[149]
		Phase III, RCT	NCT04208178, EPIK-B2	HER2+, ABC; PIK3CA+	Trastuzumab, Pertuzumab	19	PFS, OS, ORR, CBR, TTR, DOR	Active	[150]
		Phase III, RCT	NCT05063786, ALPHABET	HER2+, ABC, PIK3CA+	Trastuzumab, Fulvestrant, CT	27	PFS, OS, ORR	Active	[151]
	Taselisib (GDC-0032)	Phase Ib, non-RCT	NCT02390427	HER2+, ABC	T-DM1, Pertuzumab, trastuzumab, paclitaxel	68	MTD, DLT (Fisher’s exact test), CBR, PFS, OS (Kaplan–Meier method)	MTD, RP2D	[152]
	Inavolisib (GDC-0077)	Phase I, non-RCT	NCT03006172	PIK3CA+	Fulvestrant, Letrozole, Palbociclib, Metformin, trastuzumab, Pertuzumab	256	NA	Active	
		Phase II, RCT	NCT05306041, GeparPiPPa	HER2+, HR+, PIK3CA+	PHESGO, endocrine therapy	170	pCR, CBR	Active	[153]
		Phase III, RCT	NCT05894239, INAVO122	HER2+, PIK3CA+, ABC	PHESGO	230	OS, ORR, CBR, PFS, HRQoL	Active	[154]
AKT inhibitor									
ATP-competitive inhibitor									
	Ipatasertib (GDC-0068, RG7440)	Phase II, SAT	NCT05554380	AKT+	Paclitaxel	33	ORR, PFS, OS, DOR (Kaplan–Meier and Brookmeyer–Crowley method)	Active	
		Phase II, RCT	NCT05180006, BIS-Program	TNBC, HER2+	Atezolizumab, Bevacizumab, trastuzumab, Pertuzumab	185	pCR, biomarkers (IHC)	Active	[155]
		Phase I, SAT	NCT04253561, IPATHER	HER2+, ABC, PIK3CA+	Trastuzumab, Pertuzumab	15	pCR, PR2D, DLT, CBR, OR	Active	[156]
Allosteric inhibitor (non-ATP-competitive)									
	MK-2206	Phase I, SAT	NCT01705340	HER2+, ABC/MBC	Trastuzumab, Lapatinib, Ditosylate	60	MTD, PFS, ORR, DOR (Kaplan–Meier method)	Terminated	
		Phase II, SAT	NCT01319539	AKT+	/	12	biomarkers	No difference, pAKT decreased	[157]
		Phase I, SAT	NCT01281163	HER2+, MBC	Lapatinib, Ditosylate	4	Biomarkers	MTD (MK-2206 45 mg orally every other day + lapatinib 1500 mg orally daily)	[158]
		Phase II, SAT	NCT01277757	PI3K/AKT mutation, MBC	/	30	CBR, PFS, biomarkers, RPPA	Terminated	[159]
		Phase I, SAT	NCT01263145	MBC	Paclitaxel	34	MTD, biomarkers, RPPA (Wilcoxon rank test), correlation analyses (Pearson and Spearman)	AKT decreased	[160]
		Phase I, SAT	NCT01245205	HER2+, ABC/MBC	Lapatinib Ditosylate	28	MTD, DLT, CBR, PFS, PK	Well tolerated	[161]
		Phase I, non-RCT	NCT00963547	HER2+	Trastuzumab, Lapatinib Ditosylate	33	DLT, MTD, RP2D	CR 3%, PR 3%	[162]
	Perifosine	Phase II	NCT00054145	MBC	/	18	PFS, OS (Kaplan–Meier method)	PFS 8 weeks	[163]
mTOR inhibitor									
mTOR and its analogs									
	Rapamycin, Rapalogs	Phase III, RCT	NCT04736589	HER2+, MBC, PAM abnormal	Inetetamab, Pyrotinib, Chemotherapy	recruiting	PFS, ORR, OS, CBR, AE/SAE	Active	
		Phase II, SAT	NCT00411788	HER2+, MBC	Trastuzumab	11	CBR, ORR, DOR, biomarkers	No result published	
	Temsirolimus (CCI-779)	Phase I, SAT	NCT01155258	MBC	Vinorelbine Ditartrate	19	MTD, PFS, OS	No result published	
		Phase I/II, SAT	NCT01111825	HER2+, TNBC	Neratinib	99	ORR, CBR, DOR, PFS, OS	ORR 13.5%/29.2%, CBR 21.6%/39.6%	
		Phase I/II, SAT	NCT00699491	ABC/MBC	Cixutumumab	48	RP2D, CBR, AE, PFS (Kaplan–Meier method)	No result published	
		Phase I, non-RCT	NCT00659568	MBC, Unresectable	Metformin	28	MTD, Toxicity and safety	No result published	
		Phase II, SAT	NCT00376688	HR+, HER2+	/	31	CBR	No result published	
	Everolimus (RAD001)	Phase II, RCT	NCT00255788	MBC	/	49	Efficacy, biomarkers	HR+ subgroup CBR 12%	[164]
		Phase I, non-RCT	NCT00426556	HER2+, MBC	Trastuzumab, paclitaxel	88	ORR, BOR, PFS, OS	ORR 44%, resistance-subgroup ORR 55%	[165]
		Phase Ib, non-RCT	NCT00426530	HER2+, MBC	Trastuzumab, Vinorelbine	50	DLT, efficacy, biomarkers	ORR 19.1%, CBR 83.0%, PFS 30.7 weeks	[166]
		Phase III, RCT	NCT00567554, GeparQuinto	eBC	Paclitaxel	2600	pCR, DFS	No difference	[167]
		Phase III, RCT	NCT00876395, BOLERO-1	HER2+, ABC/MBC	Trastuzumab, paclitaxel	719	PFS, OS, ORR, CBR, OR, ECOG-PS	PFS 14.95 months, HR- subgroup PFS 20.27 months	[168,169]
		Phase II, SAT	NCT00930475	MBC	Carboplatin	15	MTD	MTD, PR 21%, CBR 43%	[170]
		Phase III, RCT	NCT01007942, BOLERO-3	HER2+, ABC/MBC	Trastuzumab, Vinorelbine	569	PFS, OS, ORR, CBR, OR, ECOG-PS, HRQoL	PFS 7 months	[171]
		Phase II, RCT	NCT01163929	HER2+	Trastuzumab, paclitaxel	Withdrawn	pCR	No result published	
		Phase II, SAT	NCT01305941	HER2+, BCBM	Trastuzumab, Vinorelbine	32	CBR, toxicity, OS, FACT	CNS CBR 30%	[172]
		Phase Ib/II, SAT	NCT01783756, TRIO-US B-09	HER2+, CNS M	Lapatinib, Capecitabine	9	CNS ORR, PFS, OS	CNS ORR 27%	[173]
		Phase Ib/II, SAT	NCT00317720	HER2+, MBC	Trastuzumab	40	MTD, CBR	CBR 34%, PFS 4.1 months	[174]
		Phase I, non-RCT	NCT00473005	MBC	Capecitabine	18	MTD, efficacy	Terminated	
	Ridaforolimus (AP23573, MK-8669), Deforolimus	Phase II, SAT	NCT00736970, 8669-009	HER2+, trastuzumab-refractory, MBC	Trastuzumab	34	ORR, CBR, safety, PFS, OS	CBR 34.3%, PFS 5.4 m, OS 17.7 months	[175]
ATP-competitive inhibitor									
	Dactolisib (BEZ235)	Phase Ib/II, RCT	NCT01471847	HER2+, Trastuzumab-Refractory	Trastuzumab	5	DLT, PFS, ORR, CBR, TTR, OS	No result published	
	GDC-0980	Phase I, SAT, randomized	NCT01254526	ABC/MBC	Paclitaxel, Bevacizumab	52	DLT, AE, PFS, PK	No result published	
RapaLink-1									

**Note**: ClinicalTrials.gov entries as of December 2014 are listed. **Abbreviations**: RCT, randomized controlled trial; pCR, pathologic complete response; ORR, overall response rate; SAT, single-arm clinical trial; DLT, dose-limiting toxicity; MTD, maximum tolerated dose; PR2D, recommended phase 2 dose; PK, pharmacokinetic; TTR, time to response; BLRM, Bayesian Logistic Regression Model; PFS, progression-free survival; CBR, clinical benefit rate; DOR, duration of response; OS, overall survival; HRQoL, Health-Related Quality of Life; IHC, immunohistochemistry; RPPA, reverse-phase proteomic arrays; AE, adverse event; SAE, serious adverse event; BOR, best overall response; OR, overall response; ECOG-PS, ECOG (Eastern Cooperative Oncology Group) performance scale; FACT, Functional Assessment of Cancer Therapy; CNS, central nervous system.

## Abbreviations

4EBP1	Eukaryotic translation initiation factor 4E-binding protein 1
ABC	advanced breast cancer
ADCC	antibody-dependent cellular cytotoxicity
ADCs	antibody–drug conjugates
BC	breast cancer
CAF	cancer-associated fibroblast
CNS	central nervous system
CR	complete response
CSC	cancer stem cell
CSF1	colony-stimulating factor 1
eBC	early-stage breast cancer
ECD	extracellular domain
EGFR	epidermal growth factor receptor
EMT	epithelial–mesenchymal transition
ER	estrogen receptor
ERAD	endoplasmic reticulum-associated degradation
FOXO	forkhead box O
GSK-3β	flycogen synthase kinase 3 beta
HER2	human epidermal growth factor receptor 2
HER2+ BC	HER2-positive breast cancer
HER2-HET	HER2–heterogeneous
HR	hormone receptor
MBC	metastatic breast cancer
MET	mesenchymal–epithelial transition
mTOR	mammalian target of rapamycin
PAM	PI3K/AKT/mTOR pathway
PI3K	phosphoinositide 3-kinase
PR	progesterone receptor
PTEN	phosphatase and tension homolog
RTK	receptor tyrosine kinase
STAT3	signal transducer and activator of transcription 3
TAM	tumor-associated macrophage
T-DM1	trastuzumab emtansine
T-Dxd	trastuzumab deruxtecan
TGFβ	transforming growth factor-beta
TKIs	tyrosine kinase inhibitors
TNBC	triple-negative breast cancer
TSC	tuberous sclerosis complex
TSPAN8	tetraspanin-8
